# MicroRNA profile of circulating CD4^+^ T cells in aged patients with atherosclerosis obliterans

**DOI:** 10.1186/s12872-022-02616-7

**Published:** 2022-04-15

**Authors:** Siwen Wang, Suiting Jiang, Ruijia Feng, Jiawei Liu, Longshan Liu, Jin Cui, Yi Shi, Junjie Ning, Benyuan Jia, Zuojun Hu, Shenming Wang

**Affiliations:** 1 Division of Vascular Surgery, The First Affiliated Hospital, Sun Yat-Sen University, No.58, Zhongshan 2nd Road, Yuexiu District, Guangzhou, 510080 Guangdong China; 2 Guangdong Engineering Laboratory for Diagnosis and Treatment of Vascular Diseases, The First Affiliated Hospital, Sun Yat-Sen University, Guangzhou, 510080 China; 3 Vascular Surgical Disease Research Center of Guangdong Province, The First Affiliated Hospital, Sun Yat-Sen University, Guangzhou, 510080 China; 4 The 8-Year Program, Zhongshan School of Medicine, Sun Yat-Sen University, Guangzhou, 510080 China; 5 Organ Transplant Center, The First Affiliated Hospital, Sun Yat-Sen University, Guangzhou, 510080 China

**Keywords:** Atherosclerosis, Atherosclerosis obliterans, Circulating CD4^+^ T cells, MicroRNAs, Molecular signature

## Abstract

**Background:**

To evaluate the specificity of the expression patterns of microRNAs (miRNAs) in circulating CD4^+^ T cells in aged patients with atherosclerosis obliterans (ASO).

**Methods:**

A comprehensive miRNA expression study was conducted using a miRNA microarray of CD4^+^ T cells isolated from peripheral blood mononuclear cells (PBMCs) of 33 patients with ASO and 24 healthy donors. A t test was used for statistical analysis, and the average linkage method was used for hierarchical clustering. The results were validated by qRT–PCR. Putative targeted pathways associated with validated miRNAs were predicted with the online software DIANA miRPath.

**Results:**

We identified 44 miRNAs based on a cutoff value of a 1.3-fold change in expression between the two groups, with 18 miRNAs showing a false discovery rate (FDR) p value < 0.05. The qRT–PCR analysis validated differences in 12 miRNAs, and 6 miRNAs were proven to be differentially expressed among three age groups (age: 35–55 years; 56–75 years; 76–95 years): the miRNAs miR-21 (p: 0.0008; 0.0009; 0.0022), miR-29b (p: 0.453; < 0.0001; < 0.0001), and miR-374b (p: < 0.0001; < 0.0001; 0.2493) showed upregulated expression in patients with ASO, while miR-142-3p (p: < 0.0001; < 0.0001; < 0.0001), miR-142-5p (p: < 0.0001; < 0.0001; < 0.0001), and miR-150 (p: < 0.0001; < 0.0001; 0.0001) showed downregulated expression in patients with ASO. The validated miRNAs participated in CD4^+^ T cell activation, proliferation, and migration pathways.

**Conclusions:**

Circulating CD4^+^ T cells in aged patients with ASO may show a distinct molecular signature. This is the first time that a distinctive, validated miRNA profile from circulating CD4^+^ T cells in atherosclerosis has been presented. This miRNA signature may be used to help elucidate the underlying mechanism of atherosclerosis. Further clinical studies and in-depth reports will contribute to identifying predictive and therapeutic targets in these patients with atherosclerosis.

**Supplementary Information:**

The online version contains supplementary material available at 10.1186/s12872-022-02616-7.

## Introduction

Lipid metabolism, inflammation, immune responses and advanced aging are major factors involved in the initiation and progression of atherosclerosis. MicroRNAs (miRNAs) have been proven to be important regulators of gene expression that post-transcriptionally modify cellular responses and function. MiRNAs are crucially involved in several vascular pathologies that show a clear association with increasing age [1]. Several studies have demonstrated that miRNAs dysregulation has a crucial role in the development of atherosclerotic disease and is involved in every step from plaque formation to destabilization and rupture [2].

Atherosclerotic cardiovascular disease is a chronic pathology related to endothelial cell (EC) damage, inflammatory cell infiltration, and intimal thickening caused by vascular smooth muscle cell (VSMC) proliferation. Atherosclerosis is a chronic immune inflammatory disease [3]. Both innate and acquired immune defenses are thought to be involved in atherosclerosis. Acquired immunity in atherosclerosis, especially the immune response of T cells to various self-antigens, such as oxidized low-density lipoprotein (oxLDL), glycoprotein, and heat shock protein (HSP), is mainly mediated by lymphocytes [4–6]. In addition, recent studies have shown that CD4^+^ T cells play a crucial role in this process [7, 8]. Elucidation of the complex gene regulatory network that controls CD4^+^ T cell immune events will substantially enhance our understanding of the development of atherosclerosis and subsequent cardiovascular diseases. These data may also help to identify some key predictive factors for the early stage of atherosclerosis to improve prediction and early diagnosis of atherosclerosis-related cardiovascular diseases.

MiRNAs are a large class of endogenous, small, noncoding RNAs, typically 20–24 nucleotides in length that regulate gene expression in cells via degradation or translational inhibition of their target mRNAs [9, 10]. Some miRNAs have been shown to regulate the adaptive immune response of CD4^+^ T cells. Rossi et al. identified distinct miRNA signatures in human lymphocyte subsets and found that miR-125b may enhance the naïve state of CD4^+^ T cells [11]. MiR-21 overexpression promoted DNA demethylation in CD4^+^ T cells and regulated self-reactive T cells in systemic lupus erythematosus [12]. MiR-29b was shown to interact with IFN-γ and induce DNA hypomethylation in CD4^+^ T cells of individuals with oral lichen planus [13]. MiR-374b-5p regulates T cell differentiation and is associated with rEg.P29 immunity [14]. Moreover, miRNA-142-3p was found to inhibit IFN-γ production by targeting RICTOR in *Aspergillus fumigatus*-activated CD4 + T cells [15]. MiR-142-5p regulated CD4^+^ T cells in human non-small-cell lung cancer through PD-L1 expression via the PTEN pathway [16]. In addition, miRNA-150 negatively regulates the function of CD4^+^ T cells through the AKT3/Bim signaling pathway [17]. CD4^+^ T cells with low miR-155 levels were less effective in EC adhesion, inducing the apoptosis of ECs and promoting the proliferation of VSMCs, which are all key events in atherosclerotic plaque formation and development [18]. Most of these miRNAs could not regulate CD4^+^ T cells in aged patients with atherosclerosis obliterans (ASO). To date, few studies have explored the CD4^+^ T cell-mediated effects on atherogenesis driven by miRNAs.

The histological features of human arteriosclerotic occlusive disease include atherosclerotic thickening, inelasticity, and arterial wall calcification. However, the expression profiles of miRNAs in the CD4^+^ T cells of patients with ASO are still unclear. This study of miRNA profiles of CD4^+^ T cells from aged patients with ASO will contribute to future studies on the diagnostic and therapeutic potential of certain miRNAs for ASO. The objective of this study was to identify miRNAs with up- or downregulated expression in ASO, which could uncover the underlying mechanisms of atherosclerosis development and pathological progression in aged patients.

## Materials and methods

### Sample selection

**Fig. 1:**
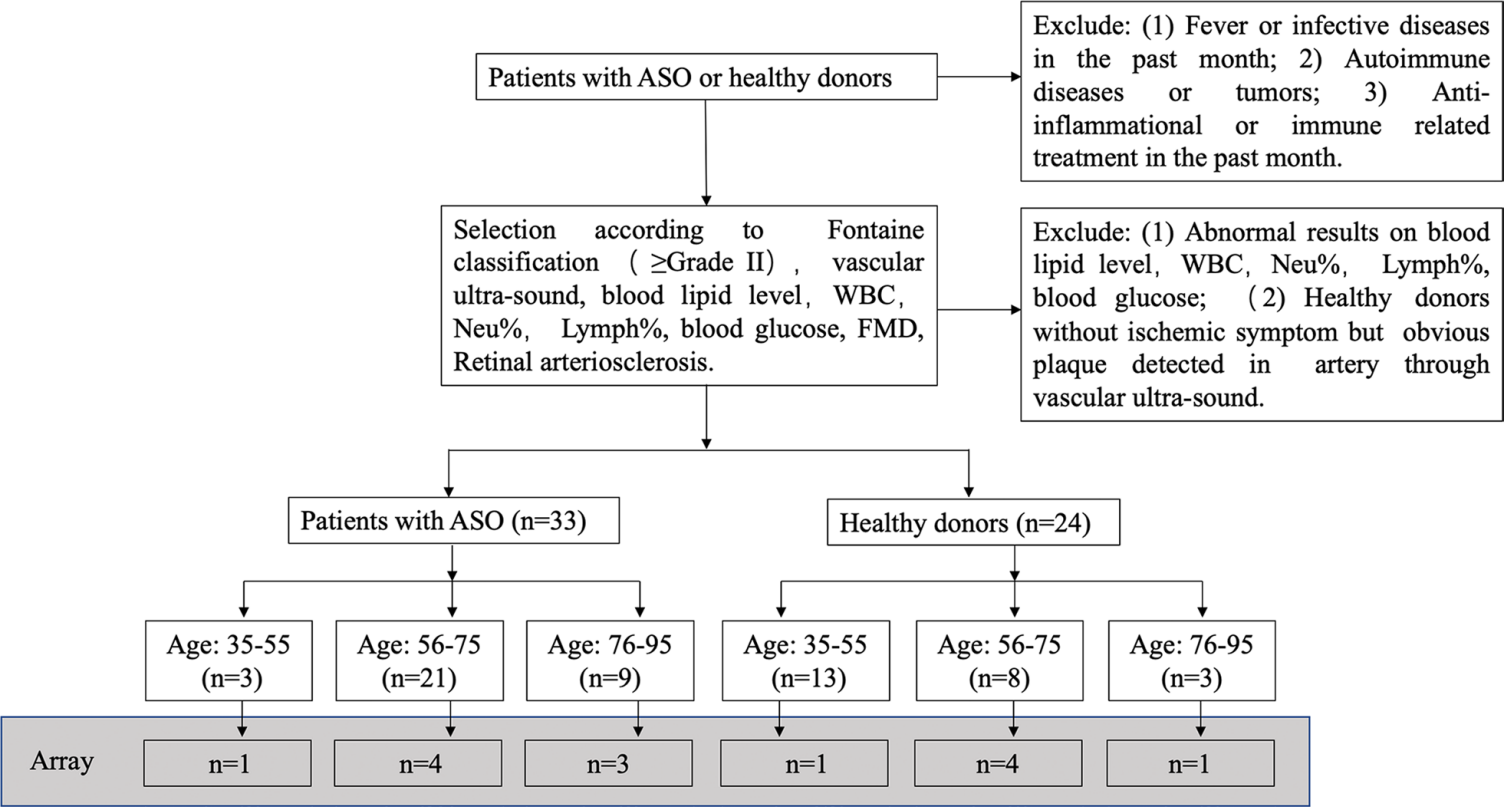
Flow diagram representing the criteria followed in the selection of the patients with ASO or healthy controls for the present study

Venous blood samples were collected from patients with ASO or healthy donors from the Chinese population at the First Affiliated Hospital of Sun Yat-sen University. As shown in Fig. 1, we divided both the patients with ASO and the healthy donors into three groups according to their age: 35–55 years old, 56–75 years old, and 75–95 years old. The included subjects met the following recruitment criteria: (1) no history of fever or infective illnesses in the past month; (2) no history of autoimmune illnesses or tumors; (3) no anti-inflammatory or immune-related treatments in the past month; (4) normal blood lipid levels and normal white blood cells (WBCs), percent neutrophils, percent lymphocytes, and blood glucose i in the past three days; and (5) manifestations of at least Fontaine grade II (patients with ASO) and no ischemic symptoms or obvious plaques in arteries through the overall body as shown by vascular Doppler examination (healthy donors). The flow-mediated vasodilation (FMD) test was performed on every patient and healthy donor. Retinal arterial atherosclerosis (stages 1–4 according to the Scheie score) was determined according to the fundus examination using direct ophthalmoscopy by an ophthalmologist blinded to the grouping of the individuals. The Ethics Committee of the First Affiliated Hospital of Sun Yat-sen University approved the use of peripheral blood mononuclear cells (PBMCs) from humans for research purposes (Ethics number: 2013A-193). All the participants provided signed informed consent to participate in this study. All methods were carried out in accordance with relevant guidelines and regulations.

After evaluation of the quantity and quality of the material, a total of 33 patients with ASO and 24 healthy controls were included (Table 1, Additional file 1: Table S1 and Table S2). Then, 14 patient samples were selected randomly for the miRNA array (Fig. 1 and Table 2).

**Table 1:** Overall characteristic of ASO patients and healthy donors included in study

Variable	ASO group	Healthy group	P value
Gender			
Male	26	16	P > 0.05
Female	7	7	
Age (years old)			
35–55	3	13	P < 0.05
56–75	21	8	
76–95	9	3	
Fontaine classification			
I	0	0	
II	10	0	
III	7	0	
IV	16	0	
Surgery	0	0	
Smoking	10	0	P < 0.01
Hepatic Lipidosis	11	0	
Hypertension	18	0	P < 0.01
Hyperlipidemia	0	0	
Diabetes	3	0	P > 0.05
Renal diseases	0	0	
Autoimmune diseases	0	0	
Infective diseases	0	0	
Immunosuppressive treatment	0	0	
Lipid-lowering agents	0	0	
Tumors	0	0	
Retinal arteriosclerosis	33	0	
FMD			
< 10%	33	0	
≥ 10%, ≤ 20%	0	24	
Blood glucose (mmol/L)			
≤ 6.0	33	24	P > 0.05
> 6.0	0	0	
WBC (× 10^9^)			
≤ 4.0	0	0	P > 0.05
> 4.0, ≤ 10.0	33	24	
> 10.0	0	0	
Lymphocytes (× 10^9^)			
≤ 1.0	0	0	P < 0.05
> 1.0, ≤ 3.3	33	24	
> 3.3	0	0	

ASO: atherosclerosis obliterans; FMD: Flow-mediated vasodilation; WBC: White blood cells

**Table 2:** Details data of included samples for microRNA microarray

Group	CD4 + cells sample ID	Gender	Age (years old)	FC	Surgery	HL	HT	Smoking	Stroke	DM	RD	HyL	Auto-ID	IST	LLA	InD	Tumors
Healthy control	CD4 + 425(1)	M	74	0	n	n	n	n	n	n	n	n	n	n	n	n	n
	CD4 + 425(3)	M	67	0	n	n	n	n	n	n	n	n	n	n	n	n	n
	CD4 + 425(5)	F	41	0	n	n	n	n	n	n	n	n	n	n	n	n	n
	CD4 + 425(6)	M	62	0	n	n	n	n	n	n	n	n	n	n	n	n	n
	CD4 + 425(7)	M	62	0	n	n	n	n	n	n	n	n	n	n	n	n	n
	CD4 + 425(13)	F	77	0	n	n	n	n	n	n	n	n	n	n	n	n	n
ASO patients	CD4 + 410(1)	F	52	4	n	n	y	n	n	n	n	n	n	n	n	n	n
	CD4 + 412(1)	M	83	4	n	n	y	n	n	n	n	n	n	n	n	n	n
	CD4 + 415(1)	M	69	4	n	n	n	n	n	n	n	n	n	n	n	n	n
	CD4 + 415(3)	M	82	4	n	n	y	n	n	n	n	n	n	n	n	n	n
	CD4 + 68(1)	M	77	4	n	n	n	n	n	n	n	n	n	n	n	n	n
	CD4 + 68(2)	M	71	3	n	n	y	y	n	n	n	n	n	n	n	n	n
	CD4 + 629(1)	F	82	3	n	n	y	N	n	n	n	n	n	n	n	n	n
	CD4 + 75(1)	M	72	2	n	y	y	n	y	n	n	n	n	n	n	n	n

ID: Identical; M: Male; F: Female; n: no; y: yes; ASO: atherosclerosis obliterans; FC: Fontaine classification; HL: Hepatic lipidosis; HT: Hypertension; DM: Diabetes mellitus; RD: Renal disease; HyL: Hyperlipidemias; Auto-ID: Auto-immune disease; IST: Immunosuppressive treatment; LLA: Lipid-lowering agents; InD: Infective disease

### CD4^+^ T cell isolation

We isolated CD4^+^ T helper cells from PBMCs according to the manufacturer’s instructions (https://www.miltenyibiotec.com/_Resources/Persistent/3c804fa07b66b63215bbacbf43387804b151d77f/SP_CD4.pdf). Venous blood samples were collected from the patients with ASO or the healthy donors at the First Affiliated Hospital of Sun Yat-sen University, and PBMCs were isolated through Ficoll centrifugation (GE Healthcare, Catalog #17144002). CD14^+^ cells were isolated from PBMCs with CD14 magnetic bead kits (Miltenyi, Catalog# 130-050-201) and FITC-conjugated anti-human CD14 antibody (BD, Catalog# 555397) according to the manufacturer’s instructions. CD4^+^ T cells were isolated from the rest of the CD14^−^ cell suspensions by positive selection with a CD4 magnetic bead kit (Miltenyi, Catalog# 130-045-101) and PE-conjugated anti-human CD4 antibody (BD, Catalog# 555347) according to the manufacturer’s instructions. Isolated CD4^+^ T cells, CD14^+^ cells and CD4^−^CD14^−^ cells were validated by FACS and frozen in liquid nitrogen for future experiments.

### Identification of cell subsets

The 200 µl CD4^+^ T cell (PE), CD14^+^ cell (FITC) and CD4^−^CD14^−^ cell subset suspensions were mixed thoroughly and centrifuged at 1500 rpm for 3 min. The cells were washed twice with 200 μl of 1:100 ice- cold perm/wash buffer (BD, Catalog# 554723) after the supernatant was discarded. Then, the cells were resuspended in the same buffer, and incubated at 4 °C for 30 min. The cells were centrifuged again, washed as described above and resuspended in 500 μl of ice-cold FACS buffer (BD, Catalog# 554723) for analysis using a FACSCalibur flow cytometer (BD).

### RNA isolation

Total RNA of CD4^+^ T cells separated from PBMCs was extracted by TRIzol (Invitrogen, Catalog# 15,596,026) and the miRNeasy Mini Kit (Qiagen, Catalog# 217,084). RNA quality and quantity were measured by using a spectrophotometer (ND-1000, Nanodrop Technologies), and RNA integrity was detected by gel electrophoresis. Some RNA samples were processed for the miRNA microarray, and other RNA samples were stored at -80 °C or processed into cDNA.

### MiRNA microarray

The 6th-generation miRCURYTM LNA Array (v.16.0) (Exiqon) was used and contains more than 1891 capture probes covering human miRNAs annotated in miRBase 16.0, as well as all viral miRNAs related to humans. In addition, this array contains capture probes for 66 new miRPlus™ human microRNAs.

### RNA labeling and array hybridization

After RNA isolation from the samples, the miRCURY™ Hy3™/Hy5™ Power labeling kit (Exiqon, Vedbaek, Denmark) was used according to the manufacturer’s guidelines for miRNA labeling. After the labeling procedure was stopped, the Hy3TM-labeled samples and Hy5TM-labeled samples were mixed pairwise and hybridized on the miRCURYTM LNA Array (v.14.0) (Exiqon) according to the array manual. A 12-Bay Hybridization System (Hybridization System—Nimblegen Systems, Inc., Madison, WI, USA) provides active mixing action and a constant incubation temperature to improve hybridization uniformity and enhance the signal. After hybridization, the slides were washed and dried following the array manual. Then, the slides were scanned using the Axon GenePix 4000B microarray scanner (Axon Instruments, Foster City, CA) [19, 20].

### Array data analysis

Scanned images were imported into GenePix Pro 6.0 (Axon) for grid alignment and data extraction. MiRNAs with two channel intensities > 0 and SNR > 1 (or one with channel SNR > 2) were chosen for further normalization. Expression data for the selected miRNAs were normalized through the lowess (locally weighted scatter plot smoothing) regression algorithm (MIDAS, TIGR Microarray Data Analysis System), which can achieve within-slide normalization to minimize the intensity-dependent differences. Between slides, normalization was performed by scale normalization (2002, Nucleic Acids Research, 30, 4 e15). After normalization, significantly differentially expressed miRNAs were identified through volcano plot filtering. Hierarchical clustering was performed using MEV software (v4.6, TIGR). The microarray dataset is publicly available at the GEO database.

### Validation by qRT–PCR

cDNA was generated from 0.5 mg of total RNA using a commercial reverse transcription kit (TaKaRa, Catalog# RR014B) according to the manufacturer’s instructions on a GeneAmp PCR System 9700 (Applied Biosystems). Then, qRT–PCR was performed with 2 ml of the generated cDNA using the protocol provided in the SYBR Green Real-time PCR Kit (TaKaRa, Catalog# RR067A) with Bio–Rad IQ5 equipment (Bio–Rad). Relative quantification was performed with 2^−(ΔCt experiment group−ΔCt control group)^. Stem–loop RT primers (TaqMan microRNA Assays, Applied Biosystems by Life Technologies, Carlsbad, California, USA, all primer details provided in the supplementary material) were used to detect miRNAs. U6 was used as a reference gene for analysis of miRNAs. Data analyses were performed via GraphPad Prism v8.00. Analysis of variance (ANOVA) was performed for multiple comparisons (p value threshold of 0.05).

### Pathway enrichment analysis and candidate gene search

DIANA miRPath pathway enrichment analysis identified the global molecular networks and canonical pathways associated with differentially expressed miRNAs (http://diana.imis.athena-innovation.gr/DianaTools/index.php?r=mirpath/index). The software is used for enrichment analysis of multiple miRNA target genes comparing each set of miRNA targets to all published Kyoto Encyclopedia of Genes and Genomes (KEGG) pathways. The pathways showing false discovery rate (FDR) p values < 0.05 were considered significantly enriched between the classes under comparison. We also searched for candidate genes using the online software miRanda, the miRNA databases miRbase (https://www.mirbase.org) and TargetScan (http://www.targetscan.org/), and previously published data. Moreover, the Gene Ontology (GO) project provides a controlled vocabulary to describe gene and gene product attributes in any organism (http://www.geneontology.org). The categories cover three domains: biological process (BP), cellular component (CC) and molecular function (MF). P values are used to denote the significance of GO term enrichment in the differentially expressed genes. The lower the p value is, the more significant the GO term is (p value ≤ 0.05 is recommended).

### Statistical analysis

Statistical significance between groups was determined by unpaired t tests, Mann–Whitney U tests, or one-way ANOVA, with Dunnett’s multiple comparison test as appropriate. P values less than 0.05 were considered significant. Correction for multiple testing was performed using FDRs [21]. Different samples in the same group were calculated with the standard error of mean (SEM); Data variability around mean of a sample were calculated with the standard deviation (SD). Correlations were evaluated by Spearman’s correlation analysis. For principal component analysis (PCA), the SPSS 25 program (SPSS, Chicago, IL, USA) was used. Comparisons between the patients with ASO and the healthy donors were made using Fisher's exact test for categorical variables.

## Results

### Identification and concentration analysis of CD4^+^ T cell subsets in the included samples

**Fig. 2:**
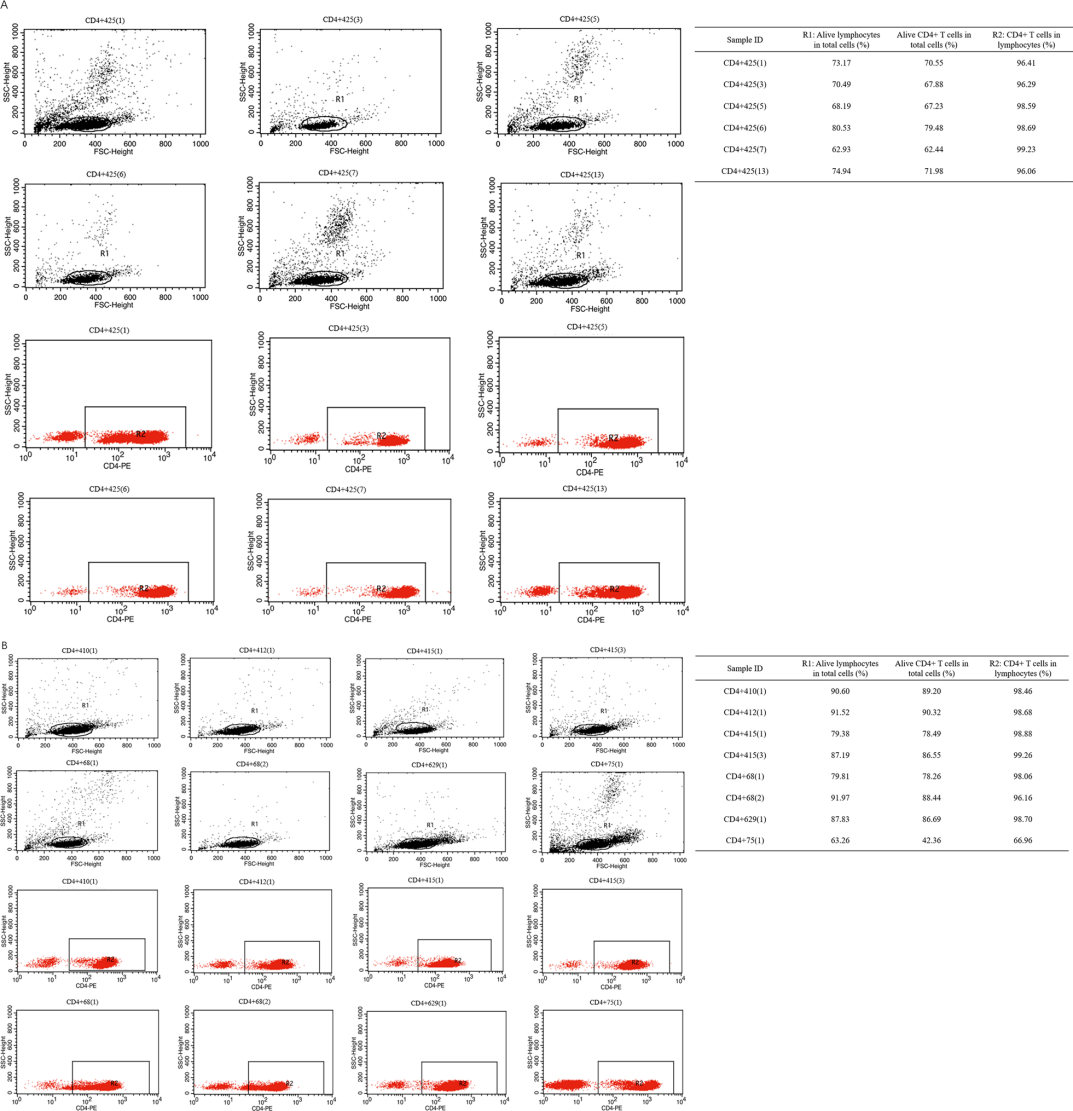
Identification of CD4^+^ cell subset ratios with FACS in the samples for the miRNA microarray (calculated with SD). **A** Six samples from the healthy control group for microarray. The proportion of cell subsets in every sample is listed on the right chart; **B** Eight samples from the ASO group for microarray. The proportion of cell subsets in every sample is listed on the right chart; R1: FACS gate for lymphocytes; R2: FACS gate for CD4^+^ T cells

All CD14^+^ monocytes, CD4^+^ T cells, and CD14^−^CD4^−^ cells in PBMCs from the donors or patients were labeled with cytokine markers and analyzed by flow cytometry. The CD4^+^ T cell ratio in CD14^+^ monocyte, CD4^+^ T cell, and CD14^−^CD4^−^ cell samples was determined by FACS cell distribution. CD4^+^ T cells were sorted from each cell subset. We determined the CD4^+^ cell proportions of the 14 samples (6 healthy donors and 8 patients with ASO) tested by the miRNA microarray. The CD4^+^ cell proportions of all 14 CD4^+^ cell samples were higher than 90% for live lymphocytes (Fig. 2). The CD4^+^ cell proportions of all 14 CD14^+^ cell samples and 14 CD14^−^CD4^−^ cell samples were lower than 10% (Additional file 1: Fig. S1 and Fig. S2). Most CD4^+^ cells were isolated from PBMC samples, and the purity of CD4^+^ cells was high for every isolated CD4^+^ cell subset, which was further subjected to miRNA microarray or candidate miRNA validation.

### MiRNA expression profiling in aged patients with ASO

**Fig. 3:**
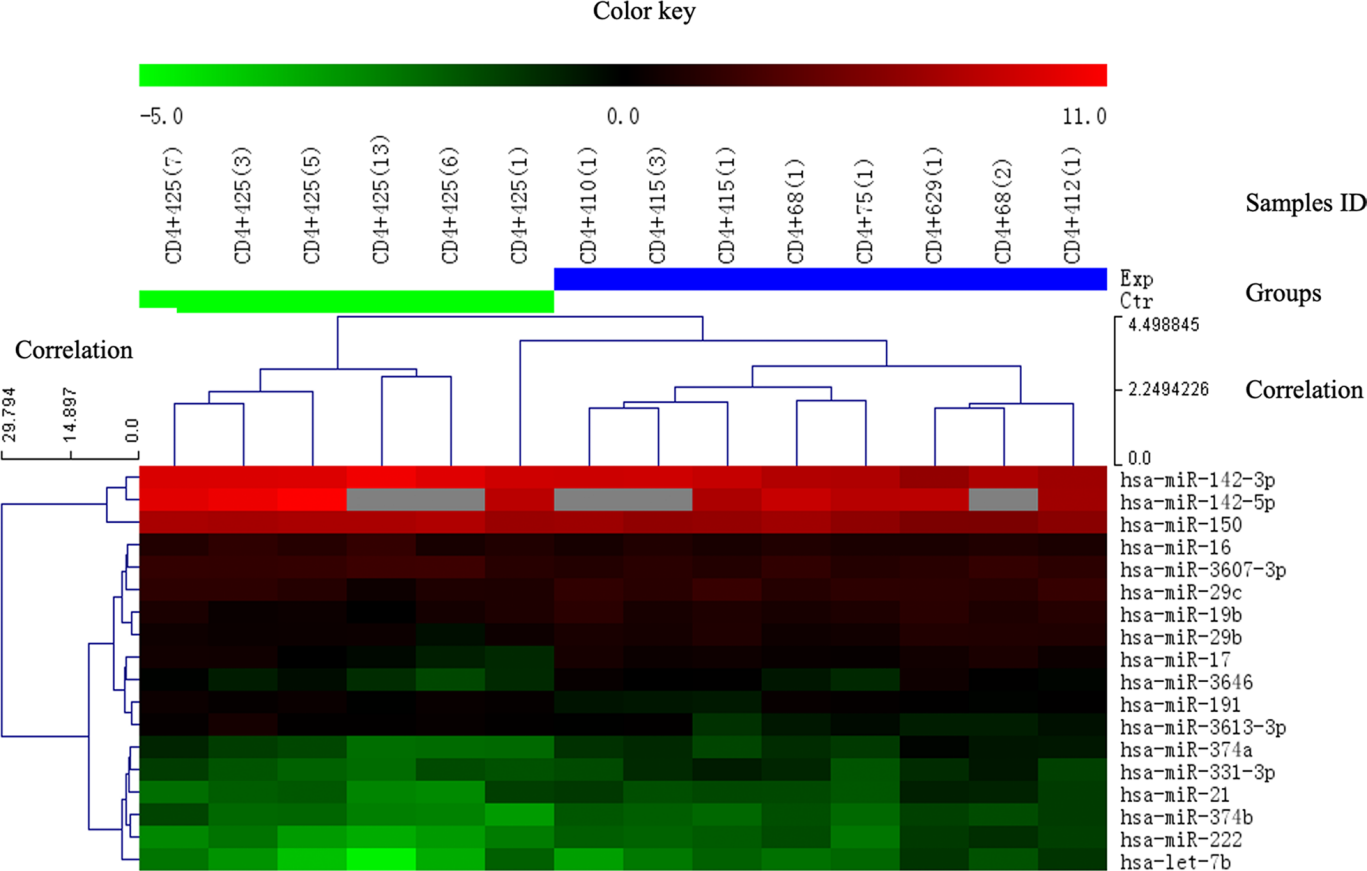
Heatmap and hierarchical clustering for different miRNAs in the Exp group vs. the ctrl group with a volcano plot (fold change ≥ 1.3, p value ≤ 0.05) (mean with SEM). The heatmap diagram shows the results of the two-way hierarchical clustering of miRNAs associated with the included ASO samples. The miRNA clustering tree is shown on the left, and the sample clustering tree is illustrated at the top. Cluster analysis arranged miRNAs and samples into groups according to their expression levels. Red indicates high relative expression, and green indicates low relative expression. Exp group: ASO group; ctrl group: healthy donor group

After the initial processing of the expression data of the 1426 miRNAs, we excluded miRNAs with low expression and reduced the number of miRNAs to a total of 108 hsa-miRNAs. Finally, we detected 44 miRNAs with a 1.3-fold change between the two groups, and after adjustment for the p value, 18 miRNAs remained significant (p value < 0.05) (Fig. 3). As shown in Fig. 3, the 7 miRNAs found to be repressed in the ASO group clustered in the top node, while the lower node shows the 11 miRNAs overexpressed in the ASO group. The expression fold change and p value of all significantly differentially expressed miRNAs are shown in Additional file 1: Tables 3S and 4S. The normalized values of the miRNAs (ratio scale-low & scale for data normalization) in every sample are listed in Additional file 1: Tables 5S, 6S, 7S, and 8S.

### Candidate miRNA selection and pathway enrichment

**Fig. 4:**
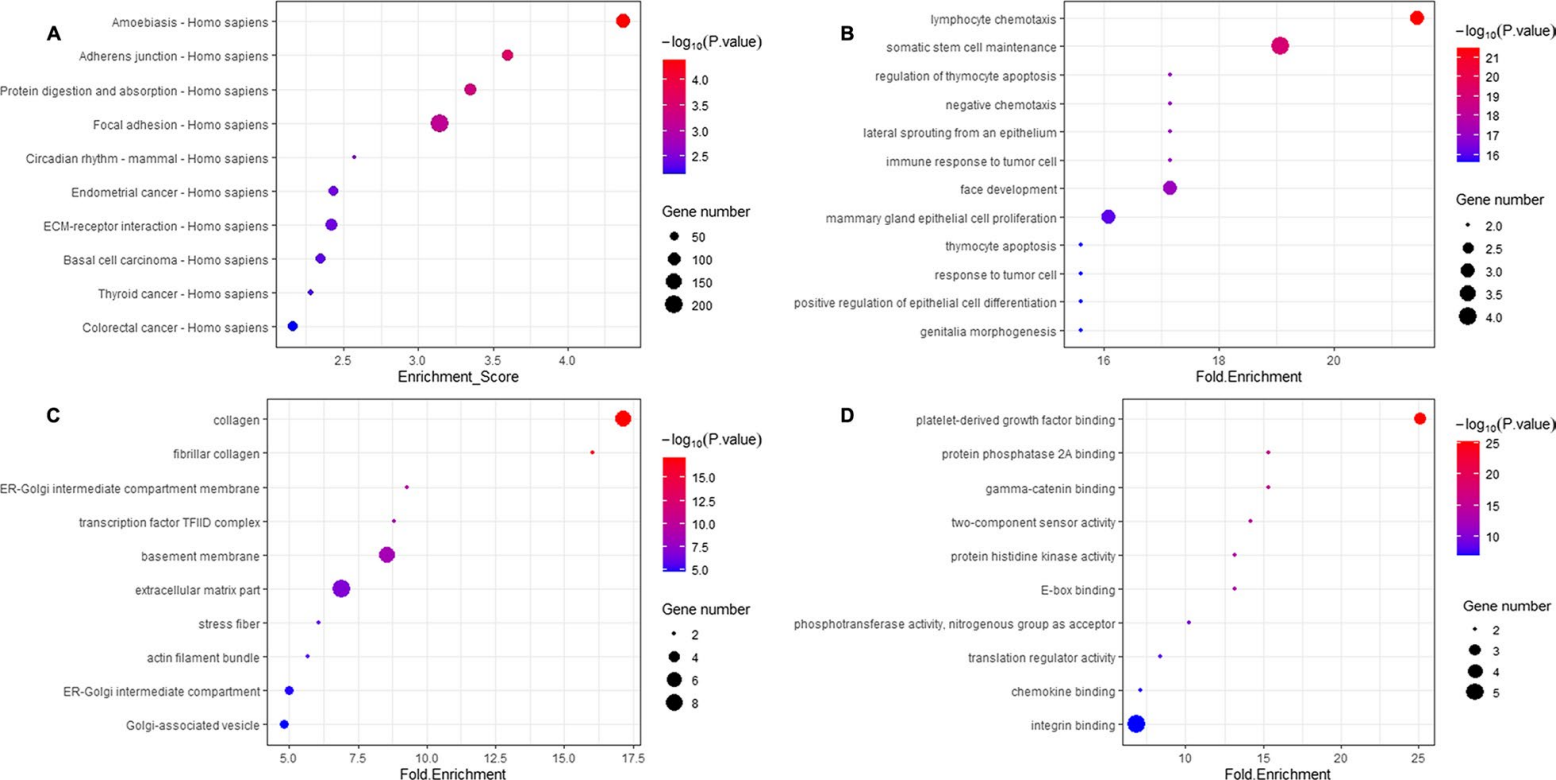
KEGG pathway enrichment analysis and GO analysis. **A** KEGG pathway enrichment analysis revealed several pathways overrepresented with FDR p value < 0.05. The bubble plot shows the top ten enrichment scores [− log10(P value)] of the significantly enriched pathways; **B**, **C**, **D** GO describes genes and gene product attributes in organisms, especially in the immune system: **B** BP; **C** CC; **D** MF. The bubble plots show the top tenfold enrichment values of the significant enrichment terms related to significant genes. KEGG pathway citation: Kanehisa, M. and Goto, S.; KEGG: Kyoto Encyclopedia of Genes and Genomes. Nucleic Acids Res. 28, 27–30 (2000). Kanehisa, M; Toward understanding the origin and evolution of cellular organisms. Protein Sci. 28, 1947–1951 (2019). Kanehisa, M., Furumichi, M., Sato, Y., Ishiguro-Watanabe, M., and Tanabe, M.; KEGG: integrating viruses and cellular organisms. Nucleic Acids Res. 49, D545-D551 (2021)

For the 18 most striking miRNAs, we performed pathway analysis and database searches to evaluate which miRNAs might be biologically relevant to inflammation and immunization and appropriate for validation. Given that miRNAs can impact a small to very large number of mRNA transcripts, the common deregulation of a group of miRNAs could potentially impact multiple biological pathways. We used DIANA miRPath v2.0 to assess pathways affected by deregulation. We assessed miRNAs from the selected hits, and KEGG pathway enrichment analysis revealed that several pathways were overrepresented, with an FDR p value < 0.05; the pathways included adhesion- and mobility-related pathways (adherent junction, focal adhesion, extracellular matrix-receptor interactions), protein digestion and absorption, cancer-related pathways (endometrial cancer, basal cell carcinoma, thyroid cancer and colorectal cancer), circadian rhythm and amoebiasis (Fig. 4A and Table 3). Based on the genes involved in the pathways related to the selected miRNAs, we used GO analysis to describe genes and gene product attributes in organisms, especially in the immune system. We found many genes related to the hits that were associated with BPs (lymphocyte chemotaxis, regulation of thymocyte apoptosis, thymocyte apoptosis, negative chemotaxis, etc.), CCs (collagen, fibrillar collagen, transcription factor TFIID complex, basement membrane, extracellular matrix part, stress fiber, actin filament bundle, etc.) and MFs (protein phosphatase 2A binding, platelet-derived growth factor binding, gamma-catenin binding, phosphotransferase activity, nitrogenous group as acceptor, translation regulator activity, chemokine binding, integrin binding, etc.) (Fig. 4B–D). Supplementary information and FDR p values associated with each pathway and GO category are shown in the Additional file 2.

**Table 3:** Significantly enriched signaling pathways associated to the validated differentially expressed miRNAs

KEGG pathway	FDR	Number of miRNAs	Putative target genes
Adherent junction	8.28 × 10^−3^	2 (miR-142-3p; miR-150)	*ACTN1, ACTN4, PVRL2, RAC1, TCF4, WASL*
Protein digestion and absorption	9.73 × 10^−3^	1 (miR-29b)	*COL15A1, COL2A1, COL3A1, COL4A1, COL4A5, COL4A6*
Focal adhesion	0.012	3 (miR-29b; miR-142-3p; miR-150)	*ACTN1, ACTN4, COL2A1, COL3A1, COL4A1, COL4A5, COL4A6, ELK1, RAC1*
Circadian rhythm	0.035	3(miR-29b; miR-142-3p; miR-150)	*ARNTL, PER3, RORB*
ECM-receptor interaction	0.035	1 (miR-29b)	*COL2A1, COL3A1, COL4A1, COL4A5, COL4A6*

KEGG: Kyoto Encyclopedia of Genes and Genomes; ECM: Extracellular matrix; FDR: False discovery rate

Finally, we focused on 12 miRNAs (7 from the repressed miRNAs and 5 overexpressed miRNAs in the ASO group) as putative candidate hits for further analysis based on their significance, previously published studies, and roles in biological pathways: hsa-miR-21, hsa-miR-374a, hsa-miR-29b, hsa-miR-19b, hsa-miR-17, hsa-miR-374b, hsa-miR-29c, hsa-miR-142-5p, hsa-miR-142-3p, hsa-miR-150, hsa-miR-16, and hsa-miR-191. Supplementary information is available in the Additional file 2.

### qRT–PCR validation for candidate miRNAs

We validated the 12 candidate miRNAs using qRT–PCR analysis of an independent sample with similar clinical characteristics. However, in the validation group, we classified the 57 samples into three subgroups (16 samples from individuals aged between 35 and 55 years, 29 samples from individuals aged between 56 and 75 years, and 12 samples from individuals aged 76–95 years) (see the clinical characteristics for all recruited patients in Table 1).

**Fig. 5:**
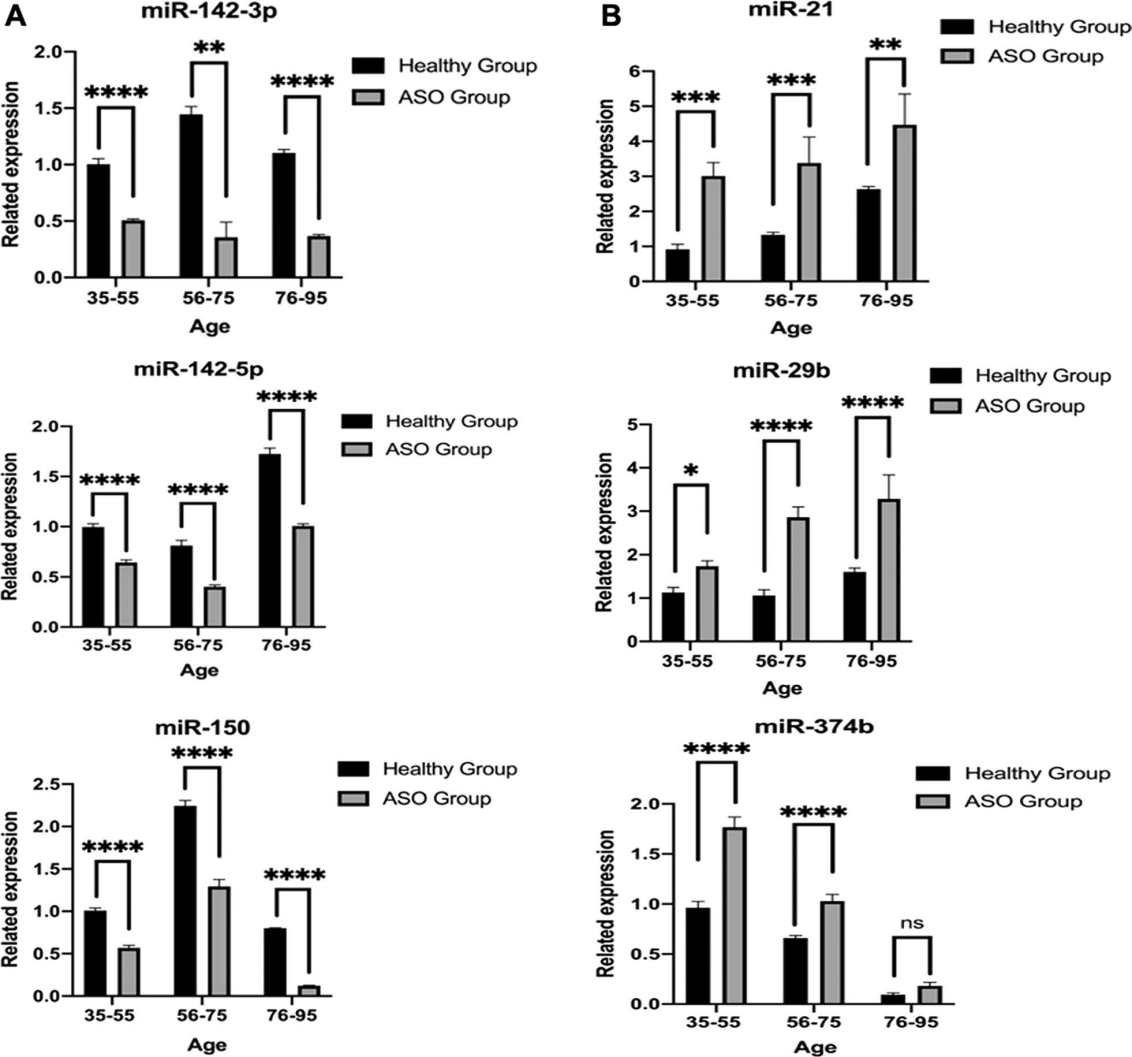
qRT-PCR validation of 6 selected miRNAs that were confirmed to have differential expression across three age groups between the patients with ASO and the healthy controls in an independent set of samples (mean with SEM). **A** Three miRNAs with downregulated expression (miR-142-3p, miR-142-5p and miR-150). **B** Three miRNAs with upregulated expression (miR-21, miR-29b and miR374b)

We did not find any evidence to suggest that the ASO group and the healthy group had different characteristics, except the symptoms and signs related to ASO in the three age groups (Tables 1 and 2). Comparing the patient samples between the ASO group and the healthy group, we confirmed that 6 of the 12 selected miRNAs showed differential expression across groups: the miRNAs with upregulated expression such as miR-21 (p value 0.0008 in the 35–55 age group; p value 0.0009 in the 56–75 age group; p value 0.0022 in the 76–95 age group), miR-29b (p value 0.0453 in the 35–55 age group; p value < 0.0001 in the 56–75 age group; p value < 0.0001 in the 76–95 age group) and miR-374b (p value < 0.0001 in the 35–55 age group; p value < 0.0001 in the 56–75 age group; p value 0.2493 in the 76–95 age group) and the miRNAs with downregulated expression such as miR-142-3p (p value < 0.0001 in the 35–55 age group; p value 0.0017 in the 56–75 age group; p value < 0.0001 in the 76–95 age group), miR-142-5p (p value < 0.0001 in the 35–55 age group; p value < 0.0001 in the 56–75 age group; p value < 0.0001 in the 76–95 age group), and miR-150 (p value < 0.0001 in the 35–55 age group; p value < 0.0001 in the 56–75 age group; p value < 0.0001 in the 76–95 age group) (Fig. 5 and Additional file 1: Fig. 3S). Notably, all of the other miRNAs tested did indeed show reduced expression or no significant difference between the ASO group and the healthy group; however, the other miRNAs still showed a significant difference between the ASO group and the healthy group in only one age group.

### Putative functional implications of the validated miRNAs

Based on the validated results of deregulated miRNAs (hsa-miR-142-3p, hsa-miR-150, hsa-miR-21, hsa-miR-29b, hsa-miR-374b), we used online software (miRanda) and miRNA databases (miRBase and TargetScan) to perform a global search for enrichment of candidate pathways and genes to establish a potential biological role. Moreover, we performed a meta-analysis on all published studies associated with the five validated miRNAs with the terms immune and/or putatively target genes. We found several enriched pathways related to T cells and atherosclerosis: adherens junctions (FDR p value 8.28 × 10^−3^), protein digestion and absorption (FDR p value 9.73 × 10^−3^), focal adhesion (FDR p value 0.012), circadian rhythm (FDR p value 0.035) and ECM-receptor interaction (FDR p value 0.035) (Table 3). All other putative target genes are available in the Supplementary information of the Additional file 2.

## Discussion

Recently, various miRNA arrays have revealed that multiple miRNAs are differentially expressed in arteries between patients with ASO and healthy donors and were related to ECs, vascular smooth muscle cells or macrophages [22–24]. Considering the importance of adaptive immunity in atherosclerosis, we performed another miRNA array with CD4^+^ T cells collected from patients with ASO. Our data revealed a differentiated miRNA signature for the group of aged patients diagnosed with ASO or atherosclerosis verified by imaging. These observations may inspire researchers to explore the possibility of detecting or delaying the development of atherosclerosis through the inhibition of CD4^+^ T cells.

Naïve CD4^+^ T cells (Th0) can differentiate into various subtypes of T helper cells, including Th1, Th2, Th17 and Treg lineages. The recognition of antigens such as oxLDL by T cell surface receptors can initiate two major different types of helper T cell immune responses, Th1 and Th2. Th1 cells participate in initiating cell-mediated immunity via macrophage activation, whereas Th2 cells are mainly involved in immune defense against external allergens and parasites [25]. Activated Th1 cells release IFN-γ to enhance antigen presentation and increase the production of TNF-α and IL-1 [26]. In contrast, Th2-related cytokines have been shown to inhibit arteriosclerosis [27]. In addition, Tregs were also shown to promote protection from atherosclerosis in apolipoprotein E knockout mice [28]. The polarization of helper T cells into different lineages is influenced by the cytokine environment. Researchers have been increasingly interested in exploring the important role of various T cell subsets in the progression of atherosclerosis. First, elucidation of the critical roles of various subsets of CD4^+^ helper T cells will help determine which T cells are “friend” rather than “foe” in atherosclerosis. Second, since the maturation of different T cell subsets is controlled by the local cytokine environment, determination of their distinct roles will likely be valuable for developing novel therapeutic strategies to limit plaque progression and subsequent rupture. Therefore, our miRNA profile results can provide clues regarding the functional miRNAs involved in the underlying mechanism of CD4^+^ T cell activation, proliferation, and migration.

The site of naïve CD4^+^ T cell activation in the early stage of atherosclerosis remains highly debated. Previous results have shown that vascular dendritic cells (DCs) contact T cells in atherosclerotic plaques. Thus, naïve CD4^+^ T cells may migrate to the plaque and are then activated. However, recent studies have indicated that antigens such as ox-LDL and HSP are recognized by antigen-presenting cells (APCs) such as DCs. Then, DCs migrate to adjacent lymph nodes and present antigens to naïve CD4^+^ T cells. Activated CD4^+^ T cells migrate to atherosclerotic plaques and affect the development of atherosclerosis [8]. Live-cell imaging of explanted aortas from CD11cYFP/ApoE–/– mice after 12 weeks of a western diet (WD: fed a high-fat and high-cholesterol diet) supported the existence of an autoantigen within the atherosclerotic plaque. In this model, antigen-experienced ApoE–/– CD4^+^ T cells were found to interact with yellow fluorescent protein (YFP) + CD11c- expressing APCs in the aortic wall [29]. These T cells are effector memory CD4^+^ T cells that have a long-term interaction with APCs in the vascular wall with slower migration speeds than T cells isolated from naïve wild-type C57BL/6 mice [30]. The major function of these interactions was to promote T cell proliferation and induce the secretion of proinflammatory cytokines (IFN-γ, TNF, and IL-17). According to these findings, a specific pathogenesis is triggered in CD4^+^ T cells in peripheral blood. Thus, some factors in CD4^+^ T cells can be screened as markers of atherogenesis. Moreover, based on the aforementioned results, changes in peripheral blood CD4^+^ T cell activation and migration could provide anti-atherosclerotic benefits.

As atherosclerotic progression of the arterial wall is initiated in newborn babies, we focused on age-related atherosclerosis in cardiovascular diseases that present some symptoms. This kind of pathological lesion may recruit specific CD4^+^ T cells and induce specific biological changes that are substantially different from physical atherogenic changes. Research on aged patients with ASO can contribute to revealing the potential mechanism typically related to CD4^+^ T cells.

MiRNAs are essential regulators of effector T cell differentiation, including T cell activation and proliferation, and can induce cytokine production. A series of recently published studies have shown that miRNAs modulate immune responses related to the pathogenesis of coronary heart disease and other atherosclerotic vascular diseases. We previously reported that miR-142-3p attenuates the migration of CD4^+^ T cells by regulating the actin cytoskeleton via RAC1 and ROCK2 in ASO [31]. Drosha- and Dicer-deficient CD4^+^ T cells showing defects in miRNA processing had poor proliferation and increased apoptosis after stimulation [32, 33]. MiR-125b was the top differentially expressed miRNA between human naïve and effector memory CD4^+^ T cell subsets. This molecule maintains the naïve state of primary human T cells via human IFNγ, IL-2Rβ, IL-10Rα and the transcriptional repressor Blimp-1 [11]. Moreover, miR-182 was recently shown to target Foxo1 to maintain T cell proliferation in the final phase of T cell expansion via IL-2, when neither TCR nor IL-2R signaling is still available [34]. MiR-146a is another miRNA that is induced by TCR stimulation and has been shown to directly target FADD to protect T cells from activation-induced cell death [35]. MiR-155 knockdown was also demonstrated to significantly hinder the ability of CD4^+^ T cells to induce EC apoptosis and to promote the growth of VSMCs. This mechanism suggests that inhibiting miR-155 in CD4^+^ T cells could slow down the formation of atherosclerotic plaques [18].

Our findings identified several significantly differentially expressed miRNAs between the ASO group and the healthy group. For example, miR-29b, which may be related to protein digestion and absorption pathways as well as focal adhesion pathways, has been reported to interact with IFN-γ and induce DNA hypomethylation in CD4^+^ T cells of individuals with oral lichen planus [36]. MiR-150, which also showed downregulated expression in patients with ASO, has been reported to negatively regulate the function of CD4^+^ T cells through the AKT3/Bim signaling pathway in acute graft-versus-host disease [37]. However, no studies have examined the relationship of miR-150 and miR-29b to atherosclerosis. Studies of miR-142-5p in CD4^+^ T cells are related to cancer or immune diseases such as multiple sclerosis. No research, except for our study on ASO, has been conducted on the relationship of miR-142-3p in CD4^+^ T cells to atherosclerosis. One study on miR-21 showed that it negatively regulates Tregs through a TGF-beta1/Smad-independent pathway in patients with coronary heart disease [38]. However, according to our qRT-PCR results, miR-21 expression was upregulated in patients with ASO. This finding suggests that miR-21 may have different mechanisms affecting CD4^+^ T cells in atherogenesis.

In conclusion, our current data indicated that miRNAs showing significantly downregulated or upregulated expression could alleviate CD4^+^ T cell-associated inflammation by affecting proliferation and migration or by altering the differentiation pattern of T lymphocytes. These changes could ultimately provide protective benefits for patients with atherosclerosis. The results that we obtained in the miRNA microarray could serve as a stepping-stone for future studies exploring the clinical potential of miRNAs in treating atherosclerosis-related diseases.

Despite the limitations of the tools currently available to manipulate miRNA biology, target aberrantly expressed miRNAs or selectively introduce miRNAs to modulate immune cell functions in atherosclerosis, our pathway and target predicted results indicated that targeting miRNAs may have important clinical potential and may represent a new direction in atherosclerosis treatment.

## Supplementary Information

**Additional file 1.** The supplementary figures and tables.

**Additional file 2.** Raw data sheets of miRNA screening and pathway analysis.

**Additional file 3.** Other supplementary material.

## Data Availability

The datasets supporting the conclusions of this article are included within the article (and its additional files) and also available from the corresponding author on reasonable request. The datasets analyzed during the current study are available in the Gene Expression Omnibus (GEO) repository with accession number GSE196698.

## Abbreviations

APC: Antigen presenting cells
ASO: Atherosclerosis obliterans
DC: Dendritic cells
EC: Endothelial cell
FDR: False discovery rate
HSP: Heat shock protein
miRNAs: MicroRNAs
oxLDL: Oxidized low-density lipoprotein
PBMCs: Peripheral blood mononuclear cells
PCA: Principal component analysis
VSMC: Vascular smooth muscle cell
