# Supplementary material for: MicroRNA profile of circulating CD4+ T cells in aged patients with atherosclerosis obliterans
Source: BMC Cardiovasc Disord. 2022 Apr 15;22:172. doi: 10.1186/s12872-022-02616-7 (PMC9013077; doi:10.1186/s12872-022-02616-7)
Supplement: Supplementary file 1 — Additional file 1. The supplementary figures and tables. [file 12872_2022_2616_MOESM1_ESM.zip › Additional file 1/Table 8S.docx]

**Table 8S: The normalized value of down regulated microRNAs (Ratio scale-Lowess & Scale for Data normalization) in Exp group samples**

| **ID** | **Name** | **CD4+410(1)** | **CD4+412(1)** | **CD4+415(1)** | **CD4+415(3)** | **CD4+68(1)** | **CD4+68(2)** | **CD4+629(1)** | **CD4+75(1)** | **Average value** |
| --- | --- | --- | --- | --- | --- | --- | --- | --- | --- | --- |
| 145798 | hsa-miR-142-5p |  | 125.771671 | 178.282924 |  | 404.367741 |  | 277.338926 | 250.001344 | 247.152521 |
| 10947 | hsa-miR-142-3p | 462.187996 | 116.829443 | 375.511131 | 501.377776 | 214.096647 | 178.200052 | 85.4846237 | 196.029627 | 266.214662 |
| 145678 | hsa-miR-150 | 114.611672 | 64.2815917 | 90.3616436 | 81.0696774 | 123.481012 | 41.7400665 | 42.0494233 | 74.8658874 | 79.0576218 |
| 148493 | hsa-miR-3613-3p | 0.97821569 | 0.80180859 | 0.50118283 | 1.0962825 | 0.72611347 | 0.66713695 | 0.67210347 | 0.86130194 | 0.78801818 |
| 10967 | hsa-miR-16 | 2.02656966 | 2.26629056 | 2.11936713 | 2.70706886 | 2.77543332 | 2.80048329 | 2.24440553 | 2.27437289 | 2.40174891 |
| 148420 | hsa-miR-3607-3p | 2.95728239 | 3.89706616 | 3.02083961 | 3.62599253 | 4.46855009 | 4.89670971 | 3.56097457 | 3.08026797 | 3.68846038 |
| 10985 | hsa-miR-191 | 0.76464219 | 1.06273632 | 0.70473376 | 0.74151627 | 1.29679285 | 0.95183798 | 1.08731498 | 1.18510542 | 0.97433497 |
