# Supplementary material for: MicroRNA profile of circulating CD4+ T cells in aged patients with atherosclerosis obliterans
Source: BMC Cardiovasc Disord. 2022 Apr 15;22:172. doi: 10.1186/s12872-022-02616-7 (PMC9013077; doi:10.1186/s12872-022-02616-7)
Supplement: Supplementary file 1 — Additional file 1. The supplementary figures and tables. [file 12872_2022_2616_MOESM1_ESM.zip › Additional file 1/Table 7S.docx]

**Table 7S: The normalized value of down regulated microRNAs (Ratio scale-Lowess & Scale for Data normalization) in Ctrl group samples**

| **ID** | **Name** | **CD4+425(1)** | **CD4+425(3)** | **CD4+425(5)** | **CD4+425(6)** | **CD4+425(7)** | **CD4+425(13)** | **Average value** |
| --- | --- | --- | --- | --- | --- | --- | --- | --- |
| 145798 | hsa-miR-142-5p | 274.647773 | 1282.97087 | 2093.33341 | NA | 902.047493 | NA | 1138.24989 |
| 10947 | hsa-miR-142-3p | 491.792726 | 716.918216 | 769.397424 | 856.880519 | 688.043341 | 1439.5691 | 827.100222 |
| 145678 | hsa-miR-150 | 103.403892 | 144.426239 | 168.104189 | 200.90494 | 165.463877 | 170.760419 | 158.843926 |
| 148493 | hsa-miR-3613-3p | 1.12136953 | 1.9732342 | 1.12345098 | 1.26800707 | 1.23160687 | 1.10413603 | 1.30363411 |
| 10967 | hsa-miR-16 | 2.77821772 | 4.24491352 | 3.29969993 | 2.07325057 | 2.85853667 | 5.02083222 | 3.37924177 |
| 148420 | hsa-miR-3607-3p | 3.41781873 | 4.60441693 | 4.93252509 | 6.43335413 | 4.73660408 | 6.24575273 | 5.06174528 |
| 10985 | hsa-miR-191 | 1.20015982 | 1.23188731 | 1.40532922 | 1.3154077 | 1.4905161 | 0.97709186 | 1.27006533 |
