# Supplementary material for: MicroRNA profile of circulating CD4+ T cells in aged patients with atherosclerosis obliterans
Source: BMC Cardiovasc Disord. 2022 Apr 15;22:172. doi: 10.1186/s12872-022-02616-7 (PMC9013077; doi:10.1186/s12872-022-02616-7)
Supplement: Supplementary file 1 — Additional file 1. The supplementary figures and tables. [file 12872_2022_2616_MOESM1_ESM.zip › Additional file 1/Table 5S.docx]

**Table 5S: The normalized value of up regulated microRNAs (Ratio scale-Lowess & Scale for Data normalization) in Ctrl group samples**

| **ID** | **Name** | **CD4+425(1)** | **CD4+425(3)** | **CD4+425(5)** | **CD4+425(6)** | **CD4+425(7)** | **CD4+425(13)** | **Average value** |
| --- | --- | --- | --- | --- | --- | --- | --- | --- |
| 11023 | hsa-miR-222 | 0.18386961 | 0.20674234 | 0.11991537 | 0.12052871 | 0.15862625 | 0.09500791 | 0.14744837 |
| 147165 | hsa-let-7b | 0.26783775 | 0.13542991 | 0.07240663 | 0.09512044 | 0.20223936 | 0.03738785 | 0.13507032 |
| 147512 | hsa-miR-21 | 0.42455129 | 0.27991395 | 0.2992047 | 0.15219663 | 0.22301566 | 0.16215916 | 0.25684023 |
| 145844 | hsa-miR-374a | 0.23874932 | 0.43486159 | 0.37949127 | 0.23109963 | 0.61043496 | 0.2242426 | 0.35314656 |
| 11040 | hsa-miR-29b | 1.55297976 | 1.40890702 | 1.46061737 | 0.81884914 | 1.60567554 | 1.49682268 | 1.39064192 |
| 42887 | hsa-miR-331-3p | 0.32779007 | 0.31819113 | 0.2609 | 0.35944051 | 0.4363071 | 0.22950351 | 0.32202205 |
| 10998 | hsa-miR-19b | 2.45446432 | 1.37121928 | 1.50546564 | 1.89870323 | 2.26691758 | 1.05827256 | 1.75917377 |
| 148481 | hsa-miR-3646 | 0.55857084 | 0.67712224 | 0.84762507 | 0.37314388 | 0.9688149 | 0.53557991 | 0.66014281 |
| 46777 | hsa-miR-17 | 0.56575732 | 1.67410873 | 0.99634019 | 0.66325705 | 1.71923087 | 0.89354206 | 1.0853727 |
| 148098 | hsa-miR-374b | 0.11865139 | 0.24084826 | 0.24961458 | 0.16866332 | 0.3869841 | 0.17682603 | 0.22359795 |
| 11041 | hsa-miR-29c | 2.51550524 | 3.96544678 | 3.12143015 | 2.5956171 | 4.06601224 | 1.52686488 | 2.96514607 |
