# Supplementary material for: MicroRNA profile of circulating CD4+ T cells in aged patients with atherosclerosis obliterans
Source: BMC Cardiovasc Disord. 2022 Apr 15;22:172. doi: 10.1186/s12872-022-02616-7 (PMC9013077; doi:10.1186/s12872-022-02616-7)
Supplement: Supplementary file 1 — Additional file 1. The supplementary figures and tables. [file 12872_2022_2616_MOESM1_ESM.zip › Additional file 1/Table 1S.docx]

**Table 1S: Details data of every individual in ASO group.**

| Patients # | Gender | | Age (Years old) | Fontaine classification | Vessel examination | Surgery | Hepatic Lipidosis | Hypertension | Smoking （years） | Stroke | Diabetes | Hyperthyroidism | Renal diseases | Hyperlipidemias | Autoimmune diseases | Immunosuppressive treatment | lipid-lowering agents | Infective diseases | Tumors | Retinal arteriosclerosis | FMD (%) | Blood glucose（mmol/L) | WBC (×10^9^) | Lymphocytes (×10^9^) | Monocytes (×10^9^) |  |
| --- | --- | --- | --- | --- | --- | --- | --- | --- | --- | --- | --- | --- | --- | --- | --- | --- | --- | --- | --- | --- | --- | --- | --- | --- | --- | --- |
| 1 | | | M | 73 | 4 | CTA, V-DU | n | y | y | 30 | y | n | n | n | n | n | n | n | n | n | y | 4.3 | 5.0 | 6.88 | 2.04 | 1.12 |
| 2 | | | F | 62 | 2 | CTA, V-DU | n | n | n | n | n | n | n | n | n | n | n | n | n | n | y | 6.4 | 4.3 | 8.08 | 1.59 | 0.44 |
| 3 | | | M | 72 | 2 | CTA, V-DU | n | y | y | n | n | n | n | n | n | n | n | n | n | n | y | 7.3 | 5.4 | 8.76 | 1.53 | 1.07 |
| 4 | | | M | 63 | 2 | CTA, V-DU | n | y | n | n | n | y | n | n | n | n | n | n | n | n | y | 5.6 | 6.0 | 7.24 | 3.12 | 0.98 |
| 5 | | | M | 60 | 4 | CTA, V-DU | n | n | y | n | y | n | n | n | n | n | n | n | n | n | y | 3.4 | 5.5 | 5.98 | 2.43 | 1.04 |
| 6 | | | M | 80 | 3 | CTA, V-DU | n | y | y | 40 | n | n | n | n | n | n | n | n | n | n | y | 5.6 | 5.0 | 3.98 | 1.26 | 0.78 |
| 7 | | | F | 52 | 4 | CTA, V-DU | n | n | y | n | n | n | n | n | n | n | n | n | n | n | y | 3.7 | 4.6 | 8.57 | 0.86 | 1.11 |
| 8 | | | M | 83 | 3 | CTA, V-DU | n | n | y | n | n | n | n | n | n | n | n | n | n | n | y | 7.9 | 5.6 | 8.66 | 0.67 | 0.93 |
| 9 | | | M | 50 | 4 | CTA, V-DU | n | y | n | 20 | n | n | n | n | n | n | n | n | n | n | y | 3.3 | 3.8 | 9.60 | 1.98 | 1.56 |
| 10 | | | M | 83 | 4 | CTA, V-DU | n | n | y | n | n | n | n | n | n | n | n | n | n | n | y | 4.7 | 5.4 | 9.31 | 0.97 | 0.9 |
| 11 | | | F | 74 | 4 | CTA, V-DU | n | n | n | n | n | n | n | n | n | n | n | n | n | n | y | 3.8 | 4.7 | 9.72 | 1.79 | 0.43 |
| 12 | | | M | 69 | 4 | CTA, V-DU | n | n | n | n | n | n | n | n | n | n | n | n | n | n | y | 2.3 | 3.8 | 8.60 | 2.03 | 0.72 |
| 13 | | | M | 75 | 4 | CTA, V-DU | n | n | y | 30 | n | n | n | n | n | n | n | n | n | n | y | 3.5 | 4.2 | 7.99 | 2.44 | 0.91 |
| 14 | | | M | 93 | 4 | CTA, V-DU | n | y | y | n | n | y | n | n | n | n | n | n | n | n | y | 2.6 | 5.9 | 7.57 | 2.52 | 0.34 |
| 15 | | | M | 65 | 3 | CTA, V-DU | n | n | n | 25 | n | y | n | n | n | n | n | n | n | n | y | 6.7 | 5.9 | 9.01 | 2.09 | 1.06 |
| 16 | | | M | 82 | 4 | CTA, V-DU | n | n | y | n | n | n | n | n | n | n | n | n | n | n | y | 3.5 | 5.3 | 7.23 | 0.61 | 0.89 |
| 17 | | | M | 77 | 4 | CTA, V-DU | n | n | n | n | n | n | n | n | n | n | n | n | n | n | y | 4.6 | 4.7 | 6.82 | 2.34 | 0.39 |
| 18 | | | M | 71 | 3 | CTA, V-DU | n | n | y | 30 | n | n | n | n | n | n | n | n | n | n | y | 7.8 | 4.9 | 6.73 | 2.32 | 0.45 |
| 19 | | | M | 70 | 4 | CTA, V-DU | n | n | n | n | n | n | n | n | n | n | n | n | n | n | y | 2.6 | 4.3 | 7.58 | 1.36 | 1.8 |
| 20 | | | F | 82 | 3 | CTA, V-DU | n | n | y | n | n | n | n | n | n | n | n | n | n | n | y | 5.0 | 4.2 | 9.30 | 2.37 | 1.13 |
| 21 | | | M | 69 | 2 | CTA, V-DU | n | n | n | n | n | n | n | n | n | n | n | n | n | n | y | 7.2 | 4.5 | 4.80 | 0.95 | 0.38 |
| 22 | | | M | 72 | 2 | CTA, V-DU | n | y | y | n | y | n | n | n | n | n | n | n | n | n | y | 8.1 | 4.5 | 5.74 | 1.43 | 0.38 |
| 23 | | | F | 74 | 3 | CTA, V-DU | n | n | n | n | n | n | n | n | n | n | n | n | n | n | y | 7.3 | 5.1 | 6.60 | 2.37 | 0.48 |
| 24 | | | M | 82 | 2 | CTA, V-DU | n | n | y | n | n | n | n | n | n | n | n | n | n | n | y | 6.5 | 4.3 | 7.10 | 1.99 | 1.03 |
| 25 | | | F | 69 | 2 | CTA, V-DU | n | y | y | n | n | n | n | n | n | n | n | n | n | n | y | 6.4 | 5.9 | 4.95 | 0.74 | 0.38 |
| 26 | | | M | 75 | 3 | CTA, V-DU | n | n | y | n | n | n | n | n | n | n | n | n | n | n | y | 7.3 | 4.6 | 6.68 | 0.82 | 0.45 |
| 27 | | | M | 71 | 4 | CTA, V-DU | n | n | n | 40 | n | n | n | n | n | n | n | n | n | n | y | 4.7 | 4.3 | 5.68 | 1.31 | 0.41 |
| 28 | | | M | 57 | 2 | CTA, V-DU | n | n | n | 30 | n | n | n | n | n | n | n | n | n | n | y | 8.6 | 4.2 | 8.93 | 2.68 | 0.62 |
| 29 | | | M | 65 | 4 | CTA, V-DU | n | y | y | 30 | n | n | n | n | n | n | n | n | n | n | y | 2.3 | 4.0 | 9.86 | 2.01 | 0.73 |
| 30 | | | M | 69 | 4 | CTA, V-DU | n | n | n | n | n | n | n | n | n | n | n | n | n | n | y | 2.4 | 4.4 | 6.54 | 0.99 | 1.46 |
| 31 | | | M | 66 | 2 | CTA, V-DU | n | n | n | n | n | n | n | n | n | n | n | n | n | n | y | 7.5 | 4.9 | 6.44 | 2.67 | 0.47 |
| 32 | | | M | 35 | 2 | CTA, V-DU | n | n | n | 15 | n | n | n | n | n | n | n | n | n | n | y | 7.4 | 4.3 | 6.64 | 2.58 | 0.61 |
| 33 | | | F | 76 | 4 | CTA, V-DU | n | y | y | n | y | n | n | n | n | n | n | y | n | n | y | 3.5 | 5.2 | 8.26 | 1.89 | 0.55 |

M: Male; F: Female; n: no; y: yes; CTA: Computer tomography angiography; V-DU: Vascular doppler ultra-sound; FMD: Flow-mediated vasodilation. WBC: White blood cells.
