# Supplementary material for: MicroRNA profile of circulating CD4+ T cells in aged patients with atherosclerosis obliterans
Source: BMC Cardiovasc Disord. 2022 Apr 15;22:172. doi: 10.1186/s12872-022-02616-7 (PMC9013077; doi:10.1186/s12872-022-02616-7)
Supplement: Supplementary file 1 — Additional file 1. The supplementary figures and tables. [file 12872_2022_2616_MOESM1_ESM.zip › Additional file 1/Table 3S.docx]

**Table 3S: 1.3 fold-up regulated microRNAs between Exp group and Ctrl group**

| **ID** | **Name** | **Foldchange**  **Exp/Ctrl** | **P-value**  **Exp/Ctrl** |
| --- | --- | --- | --- |
| 11023 | hsa-miR-222 | 2.35740992 | 0.00128344 |
| 147165 | hsa-let-7b | 2.15324646 | 0.02940041 |
| 147512 | hsa-miR-21 | 1.73152692 | 0.01249819 |
| 145844 | hsa-miR-374a | 1.7163137 | 0.01804764 |
| 11040 | hsa-miR-29b | 1.66533197 | 0.00178103 |
| 42887 | hsa-miR-331-3p | 1.63514456 | 0.01138157 |
| 10998 | hsa-miR-19b | 1.58665009 | 0.00918651 |
| 148481 | hsa-miR-3646 | 1.57164455 | 0.02735011 |
| 46777 | hsa-miR-17 | 1.51644878 | 0.02925684 |
| 148098 | hsa-miR-374b | 1.45528901 | 0.04271603 |
| 11041 | hsa-miR-29c | 1.41094694 | 0.02837319 |
