# Supplementary material for: MicroRNA profile of circulating CD4+ T cells in aged patients with atherosclerosis obliterans
Source: BMC Cardiovasc Disord. 2022 Apr 15;22:172. doi: 10.1186/s12872-022-02616-7 (PMC9013077; doi:10.1186/s12872-022-02616-7)
Supplement: Supplementary file 1 — Additional file 1. The supplementary figures and tables. [file 12872_2022_2616_MOESM1_ESM.zip › Additional file 1/Table 4S.docx]

**Table 4S: 1.3 fold-down regulated microRNAs between Exp group and Ctrl group**

| **ID** | **Name** | **Foldchange**  **Exp/Ctrl** | **P-value**  **Exp/Ctrl** |
| --- | --- | --- | --- |
| 145798 | hsa-miR-142-5p | 0.2171338 | 0.0336978 |
| 10947 | hsa-miR-142-3p | 0.32186506 | 0.00102771 |
| 145678 | hsa-miR-150 | 0.49770629 | 0.00048569 |
| 148493 | hsa-miR-3613-3p | 0.60447803 | 0.00319524 |
| 10967 | hsa-miR-16 | 0.71073604 | 0.02966552 |
| 148420 | hsa-miR-3607-3p | 0.7286934 | 0.01563664 |
| 10985 | hsa-miR-191 | 0.76715343 | 0.02008669 |
