# Supplementary material for: MicroRNA profile of circulating CD4+ T cells in aged patients with atherosclerosis obliterans
Source: BMC Cardiovasc Disord. 2022 Apr 15;22:172. doi: 10.1186/s12872-022-02616-7 (PMC9013077; doi:10.1186/s12872-022-02616-7)
Supplement: Supplementary file 1 — Additional file 1. The supplementary figures and tables. [file 12872_2022_2616_MOESM1_ESM.zip › Additional file 1/Table 6S.docx]

**Table 6S: The normalized value of up regulated microRNAs (Ratio scale-Lowess & Scale for Data normalization) in Exp group samples**

| **ID** | **Name** | **CD4+410(1)** | **CD4+412(1)** | **CD4+415(1)** | **CD4+415(3)** | **CD4+68(1)** | **CD4+68(2)** | **CD4+629(1)** | **CD4+75(1)** | **Average value** |
| --- | --- | --- | --- | --- | --- | --- | --- | --- | --- | --- |
| 11023 | hsa-miR-222 | 0.27656553 | 0.42201829 | 0.28932903 | 0.25997841 | 0.35927597 | 0.52408715 | 0.44982106 | 0.19969448 | 0.34759624 |
| 147165 | hsa-let-7b | 0.11275025 | 0.48237518 | 0.26630727 | 0.19233746 | 0.21437729 | 0.32761835 | 0.48620727 | 0.2447445 | 0.2908397 |
| 147512 | hsa-miR-21 | 0.454824 | 0.43959171 | 0.37395912 | 0.33972173 | 0.36645873 | 0.6296979 | 0.64792855 | 0.30562447 | 0.44472578 |
| 145844 | hsa-miR-374a | 0.50166253 | 0.72394527 | 0.38210252 | 0.56543427 | 0.54130022 | 0.74311868 | 0.9383206 | 0.45299819 | 0.60611028 |
| 11040 | hsa-miR-29b | 2.23433727 | 2.64804644 | 2.68214667 | 1.98866835 | 1.56919975 | 2.75367896 | 2.91045058 | 1.74051559 | 2.31588045 |
| 42887 | hsa-miR-331-3p | 0.35816452 | 0.40848192 | 0.69147387 | 0.56041633 | 0.5993641 | 0.73474688 | 0.54903778 | 0.31073548 | 0.52655261 |
| 10998 | hsa-miR-19b | 3.73790934 | 3.21681795 | 2.57784641 | 2.06161464 | 2.08706874 | 2.46156506 | 3.66348583 | 2.5232378 | 2.79119322 |
| 148481 | hsa-miR-3646 | 1.29609918 | 0.92746232 | 1.07526914 | 1.03061286 | 0.7318366 | 1.07465315 | 1.58753065 | 0.57661489 | 1.03750985 |
| 46777 | hsa-miR-17 | 2.03347887 | 1.4890322 | 1.5915259 | 1.44294951 | 1.33546781 | 2.28418674 | 1.7211593 | 1.26949651 | 1.64591211 |
| 148098 | hsa-miR-374b | 0.30259192 | 0.43994419 | 0.24103556 | 0.27611268 | 0.33944411 | 0.35123068 | 0.41322054 | 0.23961739 | 0.32539963 |
| 11041 | hsa-miR-29c | 4.5261332 | 5.27499832 | 5.54112545 | 3.59943425 | 3.14184326 | 3.47808969 | 3.85805069 | 4.04963535 | 4.18366378 |
