# Supplementary material for: MicroRNA profile of circulating CD4+ T cells in aged patients with atherosclerosis obliterans
Source: BMC Cardiovasc Disord. 2022 Apr 15;22:172. doi: 10.1186/s12872-022-02616-7 (PMC9013077; doi:10.1186/s12872-022-02616-7)
Supplement: Supplementary file 1 — Additional file 1. The supplementary figures and tables. [file 12872_2022_2616_MOESM1_ESM.zip › Additional file 1/Table 2S.docx]

**Table 2S: Details data of every individual in healthy group.**

| Patients # | Gender | Age (years old) | Vessel examination | Surgery | Hepatic Lipidosis | Hypertension | Smoking | Stroke | Diabetes | Hyperthyroidism | Renal disease | Hyperlipidemias | Auto-immune disease | Immunosuppressive treatment | Lipid-lowering agents | Infective disease | Tumors | Retinal arteriosclerosis | FMD（%） | Blood glucose（mmol/L) | WBC（×10^9^） | Lymphocytes（×10^9^） | Monocytes（×10^9^） |
| --- | --- | --- | --- | --- | --- | --- | --- | --- | --- | --- | --- | --- | --- | --- | --- | --- | --- | --- | --- | --- | --- | --- | --- |
| 1 | F | 57 | V-DU | n | n | n | n | n | n | n | n | n | n | n | n | n | n | n | 14.5 | 4.9 | 4.28 | 1.91 | 0.28 |
| 2 | M | 48 | V-DU | n | n | n | n | n | n | n | n | n | n | n | n | n | n | n | 11.6 | 5.0 | 5.62 | 2.33 | 0.40 |
| 3 | M | 55 | V-DU | n | n | n | n | n | n | n | n | n | n | n | n | n | n | n | 13.7 | 5.1 | 10.71 | 2.66 | 0.76 |
| 4 | M | 48 | V-DU | n | n | n | n | n | n | n | n | n | n | n | n | n | n | n | 11.0 | 5.4 | 9.50 | 3.42 | 0.77 |
| 5 | M | 74 | V-DU | n | n | n | n | n | n | n | n | n | n | n | n | n | n | n | 15.5 | 5.2 | 5.91 | 1.91 | 0.59 |
| 6 | M | 69 | V-DU | n | n | n | n | n | n | n | n | n | n | n | n | n | n | n | 13.4 | 5.8 | 9.20 | 1.94 | 0.77 |
| 7 | M | 35 | V-DU | n | n | n | n | n | n | n | n | n | n | n | n | n | n | n | 16.7 | 4.6 | 5.17 | 1.76 | 0.33 |
| 8 | M | 67 | V-DU | n | n | n | n | n | n | n | n | n | n | n | n | n | n | n | 17.6 | 5.2 | 5.77 | 2.08 | 0.32 |
| 9 | F | 41 | V-DU | n | n | n | n | n | n | n | n | n | n | n | n | n | n | n | 15.5 | 5.2 | 5.24 | 2.03 | 0.19 |
| 10 | M | 62 | V-DU | n | n | n | n | n | n | n | n | n | n | n | n | n | n | n | 15.3 | 4.9 | 5.87 | 1.44 | 0.52 |
| 11 | F | 40 | V-DU | n | n | n | n | n | n | n | n | n | n | n | n | n | n | n | 14.5 | 4.6 | 5.50 | 1.24 | 0.62 |
| 12 | M | 84 | V-DU | n | n | n | n | n | n | n | n | n | n | n | n | n | n | n | 16.4 | 5.6 | 5.54 | 1.59 | 0.42 |
| 13 | M | 44 | V-DU | n | n | n | n | n | n | n | n | n | n | n | n | n | n | n | 14.0 | 5.2 | 6.14 | 2.22 | 0.4 |
| 14 | M | 62 | V-DU | n | n | n | n | n | n | n | n | n | n | n | n | n | n | n | 15.9 | 4.6 | 5.99 | 2.31 | 0.29 |
| 15 | M | 35 | V-DU | n | n | n | n | n | n | n | n | n | n | n | n | n | n | n | 10.9 | 5.2 | 6.41 | 1.91 | 0.42 |
| 16 | F | 77 | V-DU | n | n | n | n | n | n | n | n | n | n | n | n | n | n | n | 13.7 | 5.6 | 6.14 | 2.37 | 0.24 |
| 17 | M | 44 | V-DU | n | n | n | n | n | n | n | n | n | n | n | n | n | n | n | 17.4 | 5.1 | 6.39 | 2.22 | 0.47 |
| 18 | M | 41 | V-DU | n | n | n | n | n | n | n | n | n | n | n | n | n | n | n | 18.5 | 4.9 | 6.84 | 2.15 | 0.47 |
| 19 | M | 70 | V-DU | n | n | n | n | n | n | n | n | n | n | n | n | n | n | n | 12.4 | 5.5 | 7.31 | 2.99 | 0.47 |
| 20 | F | 67 | V-DU | n | n | n | n | n | n | n | n | n | n | n | n | n | n | n | 13.3 | 4.8 | 9.44 | 2.41 | 0.73 |
| 21 | F | 35 | V-DU | n | n | n | n | n | n | n | n | n | n | n | n | n | n | n | 13.3 | 4.7 | 7.48 | 2.46 | 0.25 |
| 22 | M | 42 | V-DU | n | n | n | n | n | n | n | n | n | n | n | n | n | n | n | 14.4 | 5.3 | 5.55 | 2.44 | 0.25 |
| 23 | M | 78 | V-DU | n | n | n | n | n | n | n | n | n | n | n | n | n | n | n | 15.2 | 5.0 | 5.98 | 2.42 | 0.24 |
| 24 | F | 47 | V-DU | n | n | n | n | n | n | n | n | n | n | n | n | n | n | n | 16.3 | 5.0 | 5.99 | 2.03 | 0.21 |

M: Male; F: Female; n: no; y: yes; V-DU: Vascular doppler ultra-sound; FMD: Flow-mediated vasodilation; WBC: White blood cells.
