# Supplementary material for: MicroRNA profile of circulating CD4+ T cells in aged patients with atherosclerosis obliterans
Source: BMC Cardiovasc Disord. 2022 Apr 15;22:172. doi: 10.1186/s12872-022-02616-7 (PMC9013077; doi:10.1186/s12872-022-02616-7)
Supplement: Supplementary file 1 — Additional file 1. The supplementary figures and tables. [file 12872_2022_2616_MOESM1_ESM.zip › Additional file 1/Legends of supplementary figures in additional files.docx]

Legends of supplementary figures in additional files

Fig. S1: Identification of CD14+ cell subsets ratio with FACS in the samples for miRNA microarray. A. Six samples in healthy control group for microarray. The proportion of cell subsets in every sample are listed on the right chart; B. Eight samples in ASO group for microarray. The proportion of cell subsets in every sample are listed on the right chart; R1: FACS Gate for alive monocytes; R2: FACS Gate for CD14+ T cells; R3: FACS Gate for alive lymphocytes.

Fig. S2: Identification of CD14-CD4- cell subsets ratio with FACS in the samples for miRNA microarray. A. Six samples in healthy control group for microarray. The proportion of cell subsets in every sample are listed on the right chart; B. Eight samples in ASO group for microarray. The proportion of cell subsets in every sample are listed on the right chart; R1: FACS Gate for alive lymphocytes; R2: FACS Gate for CD4+ T cells.

Fig. S3: qRT-PCR validation of other 6 selected miRNAs which were confirmed to have no differential expression across three age groups between ASO patients and Healthy controls in an independent set of samples. A. Four down-regulated microRNAs (miR-17, miR-19b, miR-29c and miR374a). B. Two up-regulated microRNAs (miR-191 and miR-16).
