# Supplementary figures and images for: MicroRNA profile of circulating CD4+ T cells in aged patients with atherosclerosis obliterans
Source: BMC Cardiovasc Disord. 2022 Apr 15;22:172. doi: 10.1186/s12872-022-02616-7 (PMC9013077; doi:10.1186/s12872-022-02616-7)

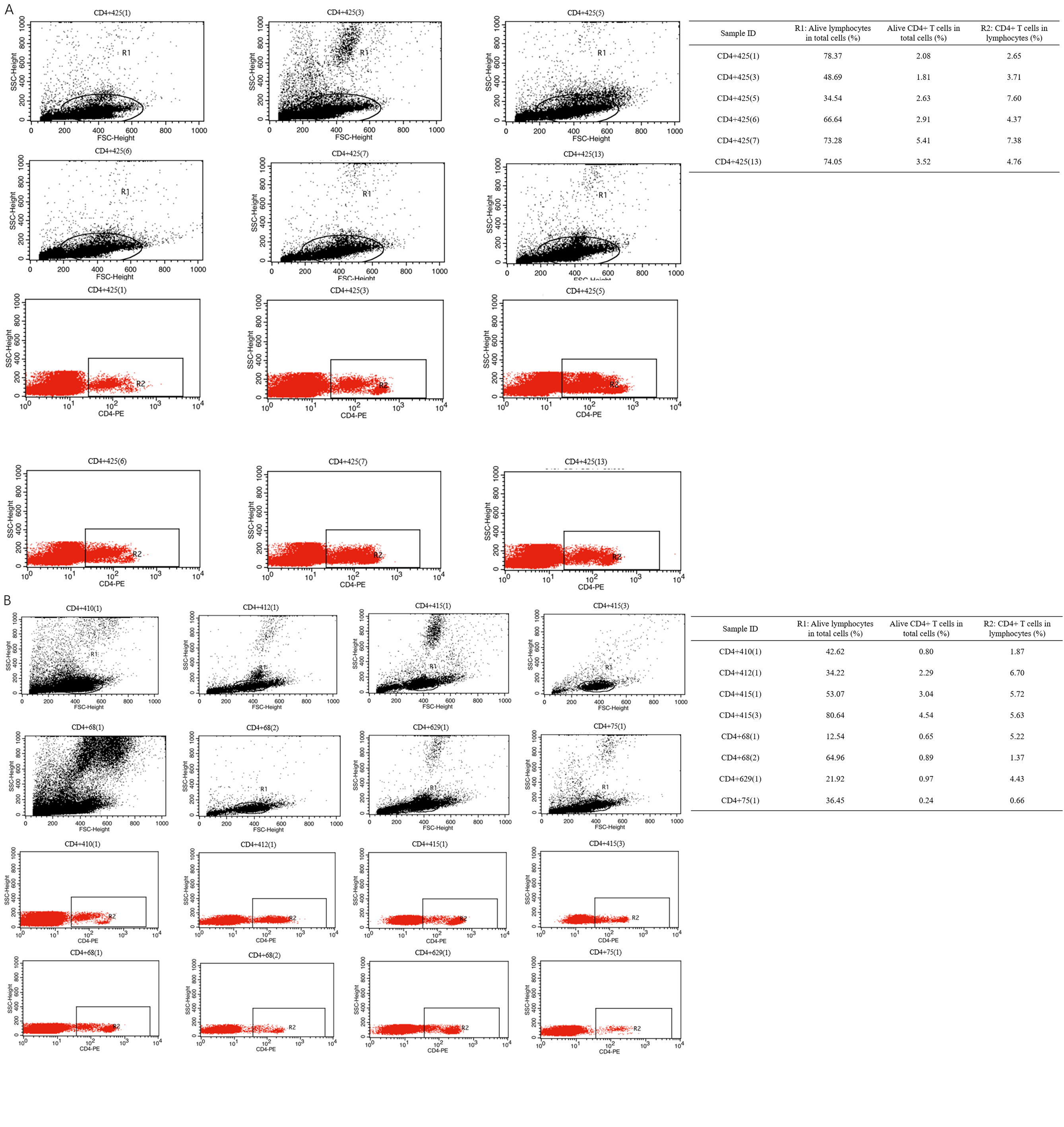

Supplement: Supplementary file 1 — Additional file 1. The supplementary figures and tables. [file 12872_2022_2616_MOESM1_ESM.zip › Additional file 1/Figure S2.tif]

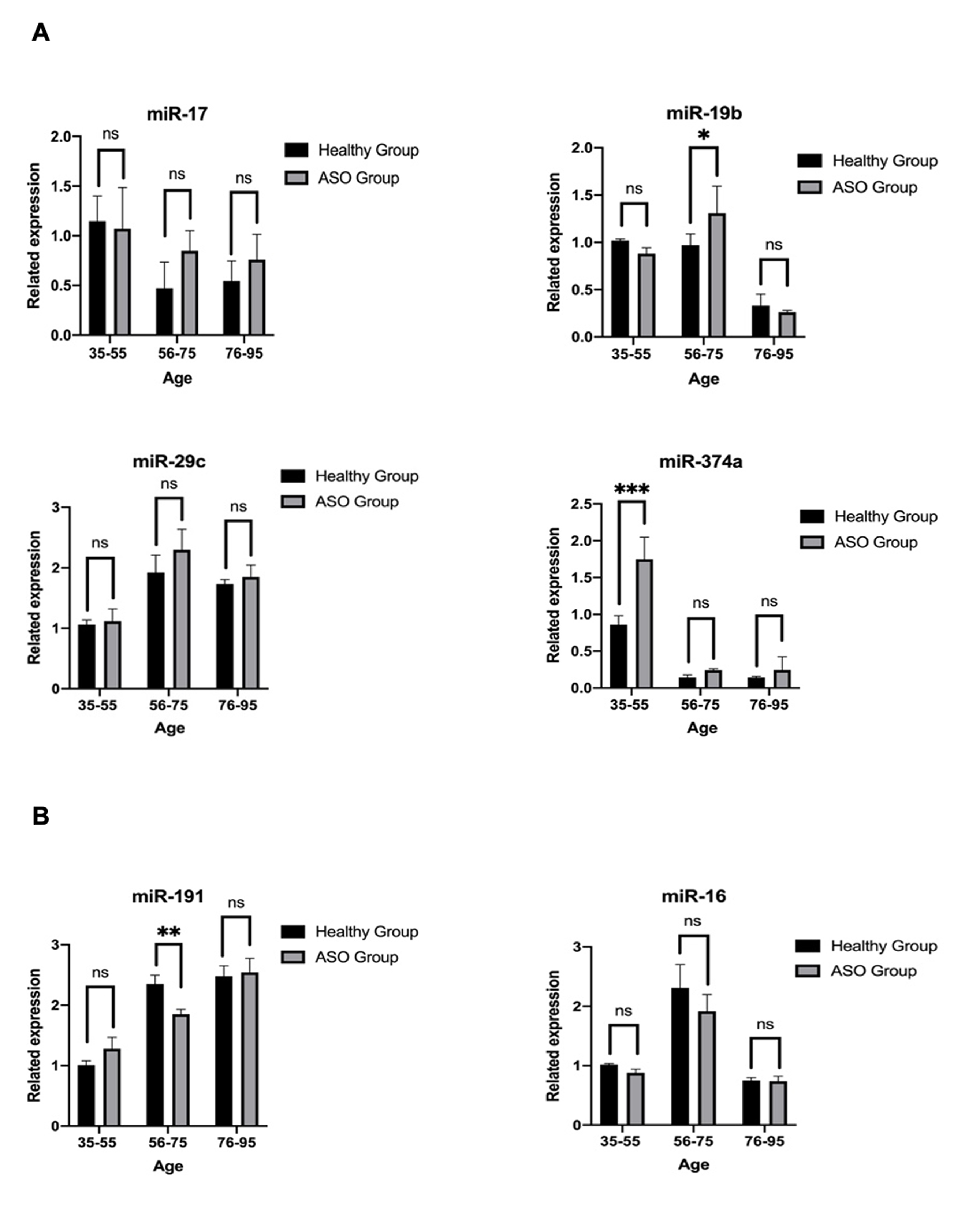

Supplement: Supplementary file 1 — Additional file 1. The supplementary figures and tables. [file 12872_2022_2616_MOESM1_ESM.zip › Additional file 1/Figure S3.tif]

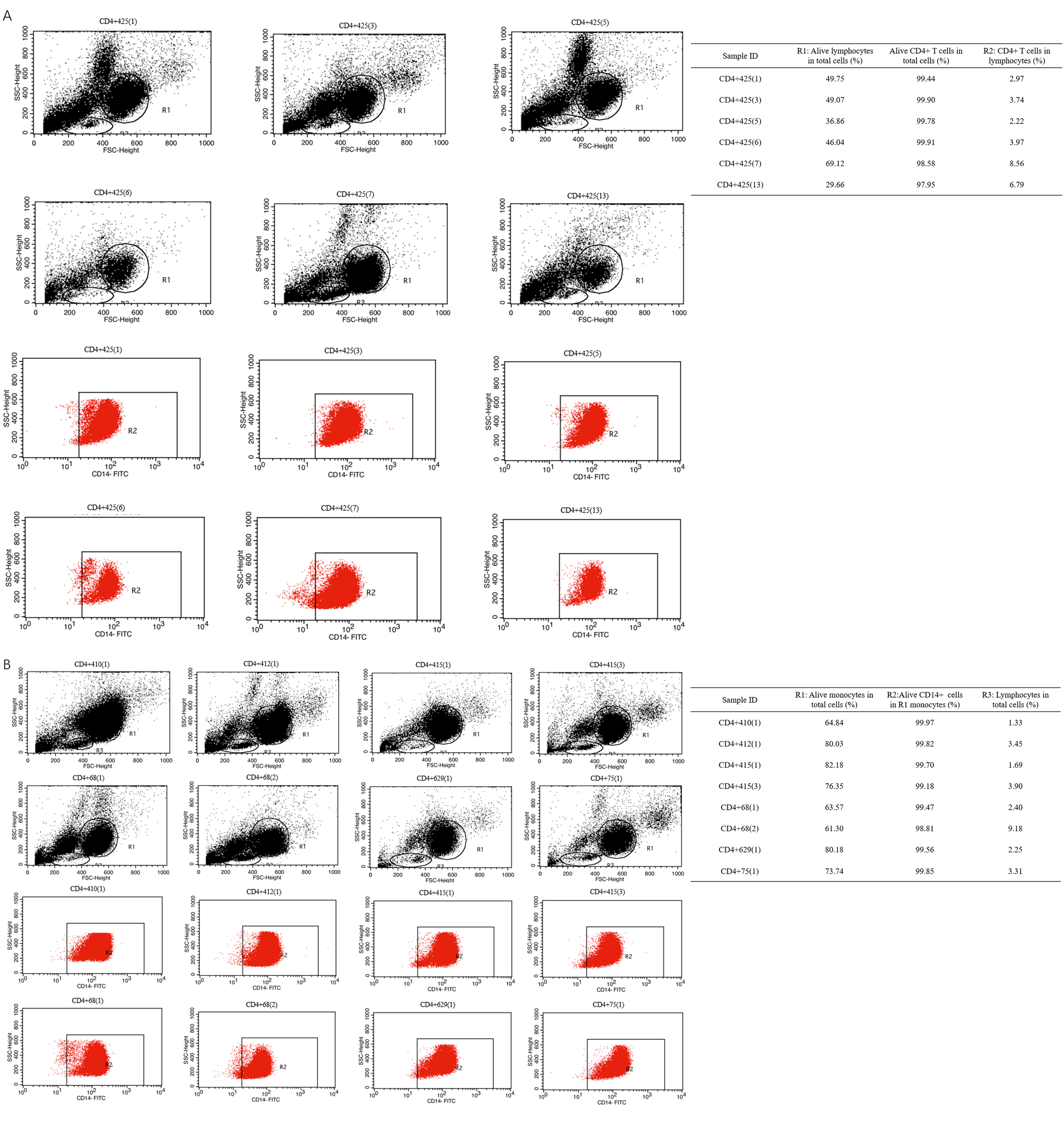

Supplement: Supplementary file 1 — Additional file 1. The supplementary figures and tables. [file 12872_2022_2616_MOESM1_ESM.zip › Additional file 1/Figure S1.tif]
