# Supplementary material for: MicroRNA profile of circulating CD4+ T cells in aged patients with atherosclerosis obliterans
Source: BMC Cardiovasc Disord. 2022 Apr 15;22:172. doi: 10.1186/s12872-022-02616-7 (PMC9013077; doi:10.1186/s12872-022-02616-7)
Supplement: Supplementary file 3 — Additional file 3. Other supplementary material. [file 12872_2022_2616_MOESM3_ESM.docx]

Complete protocol:

**1. Sample selection**

Venous blood samples were obtained from healthy donors or patients with ASO at the First Affiliated Hospital of Sun Yat-sen University from among the Chinese population. We divided both ASO patients and healthy donors into three groups according to their age: 35-55 years old, 56-75 years old, and 75-95 years old. The included subjects met the following recruitment standards: 1) no history of fever or infective diseases in the past month; 2) no history of autoimmune diseases or tumors; 3) no anti-inflammatory or immune-related treatments in the past month; 4) normal blood lipid levels and normal WBC, Neu%, lymph%, and blood glucose in the past three days; and 5) manifestations of at least Fontaine grade II (ASO patients) and no ischemic symptoms or obvious plaques in arteries through overall body by vascular Doppler examination (healthy donors). The FMD (flow-mediated vasodilation) test was performed on every patient and healthy donor. Retinal arterial atherosclerosis (stages 1 to 4 according to the Scheie score) was determined according to the fundus examination using direct ophthalmoscopy by an ophthalmologist blinded to individuals. The Ethics Committee of the First Affiliated Hospital of Sun Yat-sen University approved the use of peripheral blood mononuclear cells (PBMCs) from humans for research purposes (Ethics number: 2013A-193). All the participants provided written informed consent to participate in this study.

After evaluating the quantity and quality of the material, a total of 33 ASO patients and 24 healthy controls were included. Among them, 14 patient samples were selected for the miRNA array

**2. CD4^+^ T cells isolation**

We isolated CD4 T helper cells from PBMCs through the manufacturer’s instructions (https://www.miltenyibiotec.com/_Resources/Persistent/3c804fa07b66b63215bbacbf43387804b151d77f/SP_CD4.pdf). Venous blood samples were obtained from healthy donors or patients with ASO at the First Affiliated Hospital of Sun Yat-sen University, and PBMCs were isolated by Ficoll centrifugation (GE Healthcare, Catalog #17144002). CD14^+^ cells were isolated from PBMCs with CD14 magnetic bead kids (Miltenyi, Catalog#130-050-201) and FITC-conjugated anti-human CD14 antibody (BD, Catalog#555397) according to the manufacturer’s instructions. CD4^+^ T cells were isolated from the rest of the CD14^-^ cell suspensions by positive selection with a CD4 magnetic bead kit (Miltenyi, Catalog#130-045-101) and PE-conjugated anti-human CD4 antibody (BD, Catalog#555347) according to the manufacturer’s instructions. Isolated CD4^+^ T cells, CD14^+^ cells and CD4^-^CD14^-^ cells were validated by FACS and frozen in liquid nitrogen for future experiments.

**3. Identification of cell subsets**

The 200 µl CD4^+^ T cell (PE), CD14^+^ cell (FITC) and CD4^-^CD14^-^ cell subset suspensions were mixed thoroughly and centrifuged at 1500 rpm for 3 min. After the supernatant was discarded, washed twice with 200 μl of 1:100 ice- cold perm/wash buffer (BD, Catalog#554723), resuspended in the same buffer, and incubated at 4°C for 30 min. The cells were centrifuged and washed as above and resuspended in 500 μl of ice-cold FACS buffer (BD, Catalog#554723) for analysis using a FACSCalibur flow cytometer (BD).

**4. RNA isolation**

Total RNA of CD4^+^ T cells was extracted by TRIzol (Invitrogen, Catalog#15596026) and the miRNeasy Mini Kit (QIAGEN, Catalog#217084) according to the manufacturer’s instructions. RNA quality and quantity were measured by using a Nanodrop spectrophotometer (ND-1000, Nanodrop Technologies), and RNA integrity was determined by gel electrophoresis. Some RNA samples were processed for miRNA microarray, and other RNA samples were kept at -80°C or processed into cDNA.

**5. MiRNA microarray**

The 6th-generation miRCURYTM LNA Array (v.16.0) (Exiqon) was used, which contains more than 1891 capture probes covering all human, mouse and rat microRNAs annotated in miRBase 16.0, as well as all viral microRNAs related to these species. In addition, this array contains capture probes for 66 new miRPlus™ human microRNAs.

**6. RNA labeling and array hybridization.**

After RNA isolation from the samples, the miRCURY™ Hy3™/Hy5™ Power labeling kit (Exiqon, Vedbaek, Denmark) was used according to the manufacturer’s guidelines for miRNA labeling. One microgram of each sample was 3'-end-labeled with Hy3TM and Hy5TM fluorescent labels, respectively, using T4 RNA ligase by the following procedure: RNA in 2.0 μL of water was combined with 1.0 μL of CIP buffer and CIP (Exiqon). The mixture was incubated for 30 min at 37°C and was terminated by incubation for 5 min at 95°C. Then, 3.0 μL of labeling buffer, 1.5 μL of fluorescent label (Hy3TM or Hy5TM), 2.0 μL of DMSO, and 2.0 μL of labeling enzyme were added to the mixture. The labeling reaction was incubated for 1 h at 16°C and terminated by incubation for 15 min at 65°C. After stopping the labeling procedure, the Hy3TM-labeled samples and Hy5TM-labeled samples were mixed pairwise and hybridized on the miRCURYTM LNA Array (v.14.0) (Exiqon) according to the array manual. Then, 25 μL of the mixture from Hy3TM-labeled samples and Hy5TM-labeled samples was added to 25 μL of hybridization buffer, which was denatured for 2 min at 95°C, incubated on ice for 2 min and then hybridized to the microarray for 16–20 h at 56°C in a 12-Bay Hybridization System (Hybridization System - Nimblegen Systems, Inc., Madison, WI, USA). This system provides active mixing action and a constant incubation temperature to improve hybridization uniformity and enhance the signal. Following hybridization, the slides were washed several times using a wash buffer kit (Exiqon) and finally dried by centrifugation for 5 min at 400 rpm. Then, the slides were scanned using the Axon GenePix 4000B microarray scanner (Axon Instruments, Foster City, CA).

**7. Array data analysis**

Scanned images were then imported into GenePix Pro 6.0 software (Axon) for grid alignment and data extraction. miRNAs with two channel intensities >0 and SNR>1 (or one with channel SNR>2) were chosen for further normalization. Expression data were normalized using the lowess (locally weighted scatter plot smoothing) regression algorithm (MIDAS, TIGR Microarray Data Analysis System), which can produce within-slide normalization to minimize the intensity-dependent differences between the dyes. Between slides, normalization was performed by scale normalization (2002, Nucleic Acids Research, 30, 4 e15). After normalization, the replicated miRNAs were averaged. Differentially expressed miRNAs with statistical significance were identified through volcano plot filtering. Hierarchical clustering was performed using MEV software (v4.6, TIGR). The microarray dataset is publicly available at the GEO database.

**8. Validation by qRT**–**PCR**

cDNA was generated from 0.5 mg of total RNA using a commercial reverse transcription kit (TaKaRa,Catalog# RR014B) according to the manufacturer’s instructions on a GeneAmp PCR System 9700 (Applied Biosystems). Then, qRT–PCR was performed with 2 mL of the generated cDNA using the protocol provided in the SYBR Green Real-time PCR Kit (TaKaRa, Catalog# RR067A) with Bio–Rad IQ5 equipment (Bio–Rad). Fluorescent signals were normalized to a reference gene, and the threshold cycle (Ct) was set within the exponential phase of the PCR. Relative quantification was calculated as 2^-(ΔCt experiment group-ΔCt control group)^. Stem–loop RT primers (TaqMan microRNA Assays, Applied Biosystems by Life Technologies, Carlsbad, California, USA) were used to detect miRNAs. U6 was used as a reference gene for detecting miRNAs. Data analyses were performed via GraphPad Prism v8.00. ANOVA was performed, and Tukey’s test was used to correct for multiple comparisons (p value threshold of 0.05). The Brown-Forsythe test was used to assess the homogeneity of the variances in the different sample groups.

**9. Pathway enrichment analysis and candidate gene search**

DIANA miRPath pathway enrichment analysis was used to gain insight into the global molecular networks and canonical pathways related to differentially expressed miRNAs (http://diana.imis.athena-innovation.gr/DianaTools/index.php?r=mirpath/index). The software performs an enrichment analysis of multiple miRNA target genes comparing each set of miRNA targets to all known KEGG (Kyoto Encyclopedia of Genes and Genomes) pathways. The pathways showing FDR p values <0.05 were considered significantly enriched between classes under comparison. We also searched for candidate genes using the online software miRanda, miRNA databases miRbase (https://www.mirbase.org) and TargetScan (http://www.targetscan.org/), and previously published data. Meanwhile, the GO (Gene Ontology) project provides a controlled vocabulary to describe gene and gene product attributes in any organism (http://www.geneontology.org). The ontology covers three domains: biological process (BP), cellular component (CC) and molecular function (MF). Fisher’s exact test was used to determine if there was more overlap between the DE (differentially expressed) gene list and the GO annotation list than would be expected by chance. P values are used to denote the significance of GO term enrichment in the DE genes. The lower the p value is, the more significant the GO term (p value ≤ 0.05 is recommended).

**10. Statistical analysis**

Statistical significance between groups was determined by unpaired t test, Mann–Whitney U test, or one-way analysis of variance, with Dunnett’s multiple comparison test as appropriate. P values less than 0.05 were considered significant. Correction for multiple testing was performed using false discovery rates (FDRs)^[13]^. Correlations were evaluated by Spearman’s correlation analysis. For principal component analysis (PCA), the SPSS 25 program (SPSS, Chicago, IL, USA) was used. Comparisons between the patients with ASO and healthy donors were made using Fisher's exact test for categorical variables.

MicroRNAs primer:

| microRNA | Manufacture | Catalog # | Assay ID | miRNA sequence |
| --- | --- | --- | --- | --- |
| miR-142-3p | Thermo Fisher Scientific | 4427975 | 000464 | CUGGUACAGGCCUGGGGGACAG |
| miR-142-5p | Thermo Fisher Scientific | 4427975 | 002248 | CAUAAAGUAGAAAGCACUACU |
| miR-150 | Thermo Fisher Scientific | 4427975 | 000473 | UCUCCCAACCCUUGUACCAGUG |
| miR-21 | Thermo Fisher Scientific | 4427975 | 000397 | UAGCUUAUCAGACUGAUGUUGA |
| miR-29b | Thermo Fisher Scientific | 4427975 | 000413 | UAGCACCAUUUGAAAUCAGUGUU |
| miR-374b | Thermo Fisher Scientific | 4427975 | 001319 | AUAUAAUACAACCUGCUAAGUG |
| miR-17 | Thermo Fisher Scientific | 4427975 | 002308 | CAAAGUGCUUACAGUGCAGGUAG |
| miR-19b | Thermo Fisher Scientific | 4427975 | 000396 | UGUGCAAAUCCAUGCAAAACUGA |
| miR-29c | Thermo Fisher Scientific | 4427975 | 000587 | UAGCACCAUUUGAAAUCGGUUA |
| miR-374a | Thermo Fisher Scientific | 4427975 | 000563 | UUAUAAUACAACCUGAUAAGUG |
| miR-191 | Thermo Fisher Scientific | 4427975 | 002299 | CAACGGAAUCCCAAAAGCAGCUG |
| miR-16 | Thermo Fisher Scientific | 4427975 | 000391 | UAGCAGCACGUAAAUAUUGGCG |
